# Advances in *Chlamydia trachomatis* Vaccination: Unveiling the Potential of Major Outer Membrane Protein Derivative Constructs

**DOI:** 10.3390/microorganisms12061196

**Published:** 2024-06-13

**Authors:** Celien Kiekens, Servaas A. Morré, Daisy Vanrompay

**Affiliations:** 1 Department of Animal Sciences and Aquatic Ecology, Faculty of Bioscience Engineering, Ghent University, 9000 Ghent, Belgium; 2 Department of Genetics and Cell Biology, GROW School for Oncology and Reproduction, Maastricht University, 6229 ER Maastricht, The Netherlands; 3 Microbe&Lab BV, 1105 AG Amsterdam, The Netherlands; 4 Dutch *Chlamydia trachomatis* Reference Laboratory, Department of Medical Microbiology, Faculty of Health, Medicine & Life Sciences, Maastricht University, 6229 ER Maastricht, The Netherlands; 5 Department of Molecular and Cellular Engineering, Jacob Institute of Biotechnology and Bioengineering, Sam Higginbottom University of Agriculture, Technology and Sciences, Allahabad 211007, Uttar Pradesh, India

**Keywords:** *Chlamydia trachomatis*, STI, vaccination, subunit vaccines, MOMP

## Abstract

*Chlamydia* (*C.*) *trachomatis*, a leading cause of sexually transmitted infections (STIs) worldwide, continues to be a significant public health concern. The majority of infections are asymptomatic and, when left untreated, severe sequelae such as infertility and chronic pelvic pain can occur. Despite decades of research, an effective vaccine remains elusive. This review focuses on the potential of Major Outer Membrane Protein (MOMP)-derived constructs as promising candidates for *C. trachomatis* vaccination. MOMP, the most abundant protein in the outer membrane of *C. trachomatis*, has been a focal point of vaccine research over the years due to its antigenic properties. To overcome issues associated with the use of full MOMP as a vaccine antigen, derivative constructs have been studied. As these constructs are often not sufficiently immunogenic, antigen delivery systems or accompanying adjuvants are required. Additionally, several immunization routes have been explored with these MOMP-derived vaccine antigens, and determining the optimal route remains an ongoing area of research. Future directions and challenges in the field of *C. trachomatis* vaccination are discussed.

## 1. Introduction

The species of the *Chlamydiaceae* family are obligate intracellular pathogens of eukaryotic cells, exhibiting a characteristic biphasic developmental cycle [1]. They infect a broad range of hosts and anatomical sites [2]. This review is focused on urogenital *Chlamydia* (*C.*) *trachomatis* serovars D–K infections, the most common causes of bacterial sexually transmitted infections (STIs) in humans. Other *C. trachomatis* serovars A–C are causative agents of trachoma, the most common cause of infectious blindness in developing countries. Serovars L1–L3 belong to the lymphogranuloma venereum (LGV) biovar, causing invasive urogenital and anorectal infections [1,3]. These are beyond the scope of this review.

The global prevalence of genital *C. trachomatis* D–K was estimated by the World Health Organization (WHO) in 2020 to be 4.0% for women and 2.5% for men aged between 15 and 49 years, with 128.5 million new infections occurring worldwide [4]. However, global screening measures have not yet been implemented, so it must be emphasized that these are approximations [3]. Prevalences in certain populations have been found to be exceptionally high. For instance, in rural South Africa, 18% of the 604 women sampled between November 2011 and February 2012 tested positive for *C. trachomatis* [5]. In the United States, the Centers for Disease Control and Prevention (CDC)’s annual report indicated a significant resurgence of chlamydia cases, with 1.6 million infections reported in 2021, marking a 4.1% increase from the previous year and a return to pre-pandemic levels [6]. In Europe, data from the European Centre for Disease Prevention and Control (ECDC) indicated that chlamydia remains the most commonly reported STI in the EU. Notification rates continued to be highest among women aged 20–24 years in 2022, with an 18% increase in the rate for this population group compared to 2021 [7]. These trends highlight the importance of sustained surveillance, public education on safe sex practices, and accessible testing and treatment services to manage and reduce the spread of chlamydia.

Urogenital *C. trachomatis* infection can cause urethritis, cervicitis and salpingitis in women, and urethritis, epididymitis and epididymo-orchitis in men. Non-specific symptoms like abnormal vaginal discharge, intermenstrual bleeding, dysuria and pyuria in women, and dysuria, testicular or pelvic pain in men can develop [3,8]. However, more than 80% of infections are asymptomatic, leading to a high rate of transmission [9]. If the infection in women is left untreated and ascends from the cervix to the upper reproductive tract, severe complications like pelvic inflammatory disease (PID) can develop [3]. Approximately 20% of women infected with genital *C. trachomatis* develop PID, which can lead to chronic pelvic pain (4%), infertility (3%) and adverse pregnancy outcomes (2%) [8,10]. Moreover, the primary cause of preventable infertility and adverse pregnancy outcomes is chlamydial PID [10]. Clinical symptoms of PID are non-specific and include lower abdominal or pelvic pain, dysuria, dyspareunia, abnormal vaginal discharge or (postcoital) bleeding. Occasionally, patients also complain of chills, nausea, vomiting and fever. It is important to note that a significant proportion of women with PID never experience symptoms [3,8]. Most women with tubal infertility and serological evidence of *C. trachomatis* infection lack a history of clinical PID [11]. Persistent infections have also been associated with gynecologic tumors, increased risk of human papillomavirus (HPV) acquisition and persistence and increased human immunodeficiency virus (HIV) acquisition and transmission [9,12]. Genital infections can be vertically transmitted during vaginal delivery, causing conjunctivitis and pneumonitis in infants. This biovar can infect rectal mucosa as well, irrespective of sexual practices, possibly causing proctitis with rectal pain, discharge and bleeding [8,13]. *C. trachomatis* has the ability to become persistent in the gastro-intestinal (GI) tract in the absence of clinical disease, making it possible for women cured of genital infection to become re-infected due to auto-inoculation [14]. Untreated infections can also lead to perihepatitis or reactive arthritis, which can become chronic in some individuals [15,16]. Recent studies concluded that the infection causes obstruction of sperm transport and even causes alterations in spermatogenesis, affecting quality and quantity [17,18].

The WHO has identified sexually transmitted *C. trachomatis* as a priority for vaccine development due to the high costs associated with treatment and screening, as well as the severe long-term sequelae in women [12,19]. National control programs based on mass screening and treatment have had very little impact and their effectiveness is being debated [13,20]. Antibiotic therapy is the standard treatment for chlamydia infections. However, research indicates that this approach can sometimes lead to halted immune responses and increased susceptibility to reinfection [9,21]. Extensive research indicates that antibiotics may not fully eliminate or inhibit *C. trachomatis* infections and carry potential risks, including the development of antibiotic resistance. This highlights the need for ongoing research on alternative non-antibiotic strategies [22]. Moreover, there is a clear need for a broad-acting, protective vaccine that can prevent transmission and protect against disease [1]. MOMP has been a central focus of vaccine research because of its strong antigenic properties. However, challenges associated with using full MOMP as a vaccine antigen have led to the exploration of derivative constructs. This review provides an overview of MOMP-derived vaccine antigens that have been evaluated in animal models and tracks their evolution over the years. Additionally, we discuss the challenges these constructs face and consider potential advancements in the field of *C. trachomatis* vaccination.

## 2. Vaccine-Induced Adaptive Immune Responses against *C. trachomatis*

**Figure 1:**
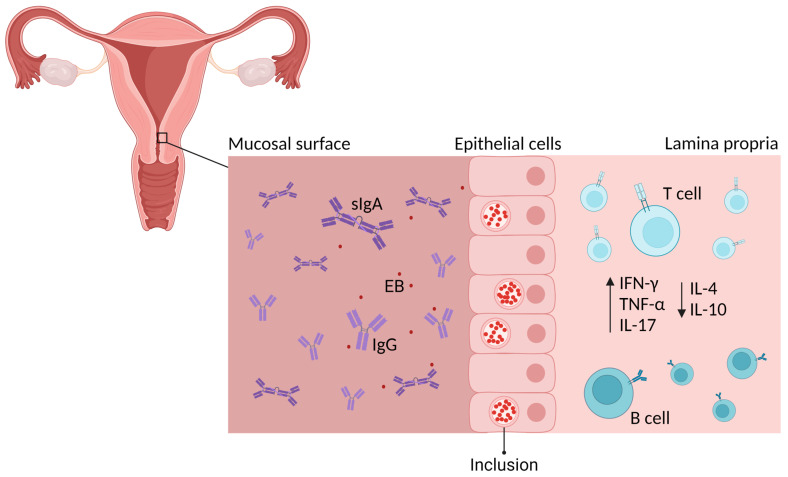
Mouse models have demonstrated that vaccine-induced adaptive immune responses against *C. trachomatis* should comprise humoral immune responses including mucosal IgG and IgA responses with neutralizing capabilities, and CMI responses, including upregulation of IFN-γ, TNF-α and IL-17 and downregulation of IL-4 and IL-10. Created with BioRender.com.

Research in mouse models identified that a successful *C. trachomatis* vaccine should likely induce (a) humoral immune responses including chlamydial-specific serum immunoglobulin (Ig)G and mucosal IgG and IgA responses with neutralizing capabilities and (b) cell-mediated immune (CMI) responses, including upregulation of interferon (IFN)-γ, tumor necrosis factor (TNF)-α and interleukin (IL)-17, and downregulation of IL-4 and IL-10 [2] (Figure 1). The primary role of mucosal neutralizing antibodies will be to reduce the initial infectious load. Once remaining bacteria infect the epithelial layer and localize intracellularly, they can be targeted by bactericidal CMI responses [23,24].

The role of humoral immune responses in *C. trachomatis* immunity has been a topic of discussion, as antibody responses have been correlated with both protection and increased risk of disease and reinfection in humans [11,13]. On the other hand, a key role for IFN-γ in the host defense against *C. trachomatis* infections has been generally accepted. Women who spontaneously cleared infections developed a CD4^+^ IFN-γ T cell response that allowed them to resist reinfection [25]. An accumulation of HLA-DR^+^ CD4^+^ and CD8^+^ T cells, primarily of memory phenotype, has been observed in the endocervix. Also, increased cytokine and chemokine levels of IFN-γ, IL-12 (T helper (Th)1 differentiation), and CX3CL1 (T cell chemoattractant) have been found in endocervical secretions [1]. A signature of central memory CD4^+^ Th17 cells has been associated with reduced *C. trachomatis* reinfection in a highly exposed cohort [26]. Such studies on the identification of the clinical correlates of protection in the context of *C. trachomatis* infections in humans are crucial for the development of effective vaccines.

Mucosal immunization is currently receiving much attention in vaccinology as most pathogens enter the body through mucosal surfaces. Development of effective vaccines able to inhibit this invasion at the mucosa is thus needed, where tissue-resident memory (TRM) T and B cells could play a key role [27]. Mucosal organs are restrictive for the entry of circulating T cells, as opposed to organs like the spleen or liver. Therefore, mucosal immunization is required to generate or permit the entry of circulating activated T cells to establish a local TRM T cell pool [28]. Studies with both conventional and humanized mice have suggested that two waves of protective memory T cells (first TRM cells, then circulating memory cells) are required for optimal clearance of *C. trachomatis* [29]. Tissue-resident T cells could directly recognize infected epithelial cells if the dendritic cells (DCs) that prime these T cells process and present the same antigens as epithelial cells [11]. However, the epithelial cell antigen repertoire was found to be discordant with the DC repertoire for *C. muridarum* infections in mice. Perhaps this could explain why it takes so long to develop natural *Chlamydia* immunity, as only 50% of *C. trachomatis* infections are resolved within 12 months. If epithelial cell antigens prove important for immunity, it is necessary to incorporate them into vaccines [30].

Another advantage of mucosal immunization is that it favors the generation of secretory (s)IgA [28]. In the vagino-cervix of humans, IgG is the predominant secreted isotype. However, sIgA, which has been proven to correlate with protection against *C. trachomatis*, has several advantages over monomeric Ig. It is more resistant to protease cleavage, it is up to 10 times more effective at neutralizing pathogens, and it has anti-inflammatory capacities, thus avoiding excessive inflammation and immune-mediated pathology [28,31,32]. Therefore, vaccination protocols for the induction of mucosal sIgA responses are the subject of intense research [12,24,33]. It has been suggested that Th17 cells are a key component in the acceleration of mucosal immunity and IgA secretion [24]. However, if serum IgG titers are sufficiently high, significant levels of mucosal IgG antibodies in the genital tracts (GTs) and eyes of non-human primates (NHPs) are detected that correlate with serum titers [34]. IgG that had transudated into the vagina of minipigs after intramuscular vaccination contributed to their protection against *C. trachomatis* [35]. The urogenital tract is more permeable to the transudation of serum antibodies than other mucosae [36]. Thus, both vaginal IgG and (s)IgA seem to have protective potential for genital *C. trachomatis* infections [24].

Mucosal immunization can target inductive sites of the mucosa-associated lymphoid tissue (MALT) to generate protective immune responses. However, the GT does not possess an organized inductive site. Therefore, alternative mucosal immunization sites like gut-, nasal-, or bronchus-associated lymphoid tissue (GALT, NALT and BALT) could be considered in *C. trachomatis* vaccination strategies, as certain inductive and effector sites of the common mucosal immune system effectively interact [28,37]. Moreover, GT vaccination depends on the host hormonal status [36]. This interaction between mucosal sites is also interesting for the development of not only genital but intestinal mucosal immunity as well, since *C. trachomatis* persistence in the GI tract occurs [13,38].

Several studies indicate that mucosal vaccination alone has immunogenic properties that are too low, so it should be conducted together with or be preceded by systemic vaccination [39,40]. However, some papers indicate protection of animals against *C. trachomatis* infection by transudate genital IgG if systemic IgG responses are high enough after systemic vaccination alone [34,35]. Also, parenterally vaccinated mice generated vaccine-derived GT tissue-resident Th1/Th17 responses post-infection similar to responses generated pre-infection by mice given simultaneous subcutaneous and intrauterine vaccinations. Both groups then showed similar vaginal IgA and IgG titers after the second infection. These results suggested that parenteral vaccination alone could be a suitable strategy against *C. trachomatis* infections, since no additional value of simultaneous mucosal vaccination against repeated infections was observed [39].

## 3. MOMP as Main Target in *C. trachomatis* Vaccination Research

Despite more than 75 years of research, no commercial vaccine for *C. trachomatis* is available today. A major barrier has been the unique biphasic life cycle of *Chlamydia* species, which challenges the immune system [34]. Traditionally, elementary bodies (EBs), either live attenuated or inactivated, were the vaccine antigens of choice. However, research has shifted towards subunit vaccination strategies and nucleic acid vaccine platforms [2]. A general comparison of the different vaccination strategies is given in Table 1.

**Figure 2:**
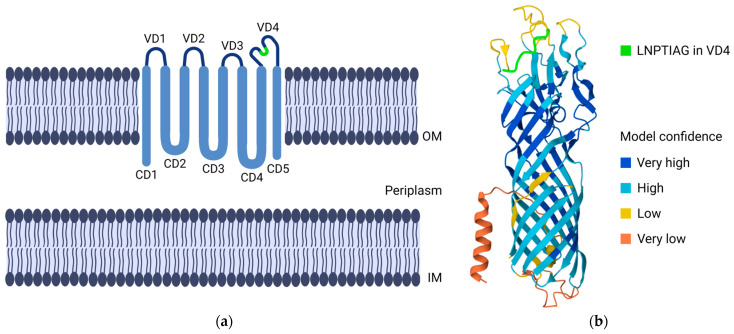
(**a**) Schematic representation of MOMP, located in the outer membrane (OM) of *C. trachomatis*. Comparative analysis of amino acid sequences shows that MOMP is organized into four surface-exposed VDs, separated by five hydrophobic, membrane-spanning CDs. (IM—inner membrane); (**b**) AlphaFold-predicted structure of MOMP from *C. trachomatis* SvE/Bour (accession number AF-P17451-F1), demonstrating the β-barrel porin structure. LNPTIAG is a conserved species-specific epitope in VD4, indicated in green in both figures. Created with BioRender.com.

MOMP has emerged as the most promising vaccine antigen candidate, evident by being the target antigen in more than 50% of protein-based vaccine trials in *Chlamydia* research [2,42]. MOMP is an important mediator of host cell attachment and entry. It is an immunodominant antigen and is expressed throughout the entire developmental cycle, emphasizing its potential as a vaccine antigen [43]. Comparative analysis of the amino acid sequences shows that MOMP is organized into four variable domains (VDs), separated by five conservative domains (CDs), schematically represented in Figure 2a [44,45]. MOMP is a surface-exposed, transmembrane protein (~40 kDa) with structural and functional β-barrel porin properties [43]. The AlphaFold-predicted 3D structure (accession number AF-P17451-F1) of MOMP, which illustrates this β-barrel porin architecture, is shown in Figure 2b [46,47]. MOMP is a cysteine-rich protein that makes up about 60% of the total outer membrane, giving structural integrity to EBs by disulfide bonds, as they contain reduced levels of lipopolysaccharide (LPS) [1,42]. Binding of EBs to a host cell is a two-step process, starting with a reversible, low-affinity electrostatic interaction followed by an irreversible, high-affinity binding mediated by MOMP binding to the host cell mannose receptor, among others [1].

DNA sequencing and computational analysis have indicated that MOMP is highly likely to be responsible for the successes observed in whole-cell vaccine trials [2]. The first MOMP-based vaccination trials were performed with native MOMP (nMOMP), which gave similar levels of protection to live EB vaccines in mice when adjuvanted with CpG and Montanide ISA 720 [48]. However, extraction of nMOMP from *Chlamydia* is laborious, time-consuming and relatively costly, making this strategy unfeasible [2,37]. Since 1992, recombinant MOMP (rMOMP) vaccine trials have emerged. The first successful rMOMP vaccine was reported by Berry et al. (2004) using a cholera toxin and CpG as adjuvants [49]. Long-term protection in mice against vaginal shedding and infertility following *C. muridarum* genital infection after rMOMP vaccination has been observed [50]. However, results vary, which is mostly attributed to the fact that the rMOMP vaccine antigen does not mimic the native structure, as predicted in Figure 2b [51]. Specific tertiary structures of MOMP are essential for its function and antigenicity. Acquiring this native structure, complete with conformationally relevant epitopes, is challenging due to the cysteine-rich structure of MOMP [52]. Also, the membrane-bound nature of the protein makes it more difficult to express and purify MOMP in soluble form in recombinant systems [37]. Achieving the correct glycosylation and post-translational modifications is challenging outside the natural host as well [53]. Efforts to improve the rMOMP folding demand altered gene sequences, vigorous detergent extraction, and long purification processes [37]. Producing large quantities of correctly folded MOMP that retains its immunogenic properties in a cost-effective manner is therefore a challenge for large-scale vaccine production. Additionally, only homotypic immunity is observed in an NHP model following MOMP vaccination [54]. Type-specific responses to MOMP can dominate, blocking the neutralizing ability of species-specific antibodies [55]. Moreover, issues with suppressive immune responses by the intact protein [56] have prompted the design of vaccines consisting of derivative constructs of MOMP. The goal is to design a vaccine that directs the immune response towards protective epitopes without evoking a strong response to non-protective or even counterproductive epitopes.

Nevertheless, efforts to develop expression systems that improve the folding and solubilization of MOMP are ongoing [37]. Studies on the structures of MOMP-derived constructs suggest that their success as vaccine antigens still relies on their conformation [57,58]. Nucleic acid vaccine platforms bypass the need to synthesize proteins in vitro and prompt Th1-skewed responses, which makes them interesting in this MOMP-based *C. trachomatis* vaccine context [1]. Some DNA plasmid platforms have been investigated for MOMP-derivative constructs [34,52,59,60,61]. The use of mRNA for *C. trachomatis* vaccination has not yet been extensively researched [1]. Self-amplifying mRNA with a MOMP insert, complexed in cationic adjuvant formulations, induced MOMP-specific IFN-γ-dominated CMI responses and antibody responses in mice, underlining its potential [62].

## 4. MOMP-Derivative Vaccines Evaluated in Animal Models

There has been significant interest in identifying specific MOMP epitopes, particularly by mapping regions detected by specific (neutralizing) antibodies and T cells from infected humans [56,63,64,65,66]. Identifying epitopes that provide cross-protection between different serovars has gained attention. In this context of cross-serovar protection, serotyping has led to the separation of *C. trachomatis* serovars into two major groups and an intermediate group, later divided into two minor groups: the B complex (B/Ba, D/Da, E, L1 and L2), the C complex (A, C, H, I/Ia, J/Ja), the B-related complex (F, G/Ga), and the C-related complex (K, L3), mainly based on the immunogenic properties of MOMP [67,68,69]. Molecular techniques offer direct and more accurate alternatives for serotyping. Results from genotyping based on the *ompA* gene (encoding MOMP) are highly concordant with traditional MOMP-based serotyping results. However, strains identified through molecular approaches should be designated as genotypes [70].

Detailed genetic and immunological knowledge of MOMP has led to many attempts to design a MOMP-derivative vaccine antigen containing antigenic determinants, overcoming immunodominance while still generating a broad immune response [52,71]. Additionally, the in silico prediction of MOMP epitopes has led to the design of potential multi-epitope MOMP vaccines [42,52,59,72,73,74]. These constructs often require an antigen delivery system or accompanying adjuvant to generate adequate co-stimulation and a favorable cytokine environment [2,75]. Th1-polarizing antigen delivery systems or adjuvants are of particular interest. Hansen et al. (2008) vaccinated mice with nMOMP with either a Th1-promoting cationic adjuvant formulation (CAF)01 or a Th2-promoting alum adjuvant. The former significantly reduced vaginal *Chlamydia* load post-infection when compared to control mice. Protection was CD4^+^ T cell- but not MOMP-conformation-dependent [76]. Combining several delivery systems or adjuvants to induce a more diverse immune response has also been a research topic [27,34,36,38,77].

### 4.1. Antigen Constructs

Table 2 provides an overview of the MOMP-derived antigen constructs that have been evaluated in animal models to date, along with the observed responses. A more detailed overview is given in Supplementary Table S1. The significance of the VD4 region of MOMP was already recognized during the first vaccination trial in animals with a MOMP-derivative construct [78], where rabbits were systemically vaccinated with a fragment of MOMP (AA_273–333_) linked to *Schistosoma japonicum* glutathione S-transferase. More than 60% of the papers on MOMP-derivative vaccine constructs tested in animal trials focus on VD4. This region is of particular interest because it is the largest and most antigenically complex of the VDs. It induces neutralizing antibodies and contains a highly conserved species-specific epitope LNPTIAG (Figure 2), possibly situated within a hydrophobic cleft [78,79]. However, findings indicate that despite this species-specific epitope, a vaccine based on a VD4 sequence might only offer protection against certain *C. trachomatis* serovars, particularly those within the same complex. Cloned VD4 fragments from the different complexes would be needed to achieve cross-protection against all serovars [78,79,80]. Protein structure prediction models and experimental evidence suggest that the VD4-conserved epitope conformation may vary during the course of infection, depending on the environmental conditions. The epitope is likely involved in the porin function of MOMP, explaining its high conservation in an otherwise variable region. Subunit vaccines containing this conserved epitope may benefit from optimization to elicit antibodies to specific constrained conformations of the epitope [58].

Early research on MOMP-derivative constructs focused on developing (neutralizing) antibody responses [78,79,80,81,83], despite suggestions as early as 1990 that antibody responses alone might not provide complete protection. Effective neutralization may require conformation-dependent B-cell epitopes, which are difficult to generate with peptide or genetic technology [84]. Therefore, subunit constructs including T cell epitopes were needed. Su and Caldwell (1993) fused an A8 peptide (AA_106–130_) from serovar (Sv)A containing a Th epitope with the VD4 region (AA_293–309_) from SvB, enhancing antibody responses in more mouse strains than VD4 alone [80]. This finding stimulated further research wherein peptides from the VD4 region were fused with other regions such as VD1 [90,91], VD2 [92], universal T cell helper peptides [73,82] or *C. trachomatis*-specific T cell epitope-containing domains [23,75,85,86,87]. Fusions of VD1 and VD4 showed that the immunogenicity of epitopes depends on their position within chimeric peptides [91]. Co-administration of universal T cell helper peptides encapsulated in liposomes did not improve immune responses to VD4 [82], while adding a universal Th1 PADRE epitope to a fusion construct of MOMP_370–387_ from SvE and Hepatitis B virus core antigen (HBcAg) enhanced immunogenicity [73]. The TINKP peptide, a 12 amino acid sequence on the edge of VD3, has been studied as a T cell helper peptide as well, since 80% of naïve human volunteers generated proliferative T cell responses, indicating that it is a promiscuous, non-haplotype-restricted peptide, conserved among all *C. trachomatis* serovars. While responses were disappointing when administered alone [84], combining it with a VD4 peptide showed promising results [75]. The MOMP_278–370_ region (M278), containing VD4 and the C-terminal region, has also gained interest as a vaccine antigen candidate. When administered unadjuvanted, antibody responses were lower than for rMOMP-vaccinated mice, but isotypes suggested a mixed Th1/Th2 response, whereas rMOMP vaccination resulted in a Th2-dominated response [86]. Encapsulation of M278 in self-adjuvanting PLA-PEG nanoparticles increased T cell cytokine and serum antibody responses [85], achieving significant protection against homologous challenge in mice, as indicated by reduced bacterial load [87]. The Zhang research group has extensively studied multi-epitope constructs of MOMP based on epitope prediction data using the SYFPEITHI program, which is based on more than 7000 peptide sequences known to bind to both class I and class II MHC molecules. Chimeric constructs combining several MOMP regions have demonstrated immunologic advantages over single-unit constructs [59,72].

The Statens Serum Institut has conducted extensive research on the VD4 region and its surrounding membrane anchor (extVD4) as vaccine antigens, due to their numerous T and B cell epitopes. Their multivalent Hirep constructs, which combine extVD4 peptides from SvD, SvE, SvF (and SvG) generated amplified antibody responses compared to rMOMP or VD4 alone [23,94]. The surrounding amino acids significantly affect antibody responses, presenting a challenge for vaccine design, as Hirep1 (extVD4^D^-extVD4^E^-extVD4^F^) vaccination in mice induced a strong antibody response towards the neutralizing epitope LNPTIAG, while extVD4^F^*4 vaccination skewed responses to a non-neutralizing epitope slightly upstream in the sequence. Hirep1 vaccination, as opposed to extVD4^F^*4 vaccination, induced significant protection against infection in mice, in both short- and long-term experiments [94]. Combining Hirep constructs with other MOMP regions (CTH522) and other *C. trachomatis* antigens (CTH93) to promote cell-mediated immunity resulted in strong CMI responses alongside high antibody titers from the repeated extVD4 regions [28,35]. Boje et al. (2016) mapped T cell responses and found multiple vaccine-induced T cell epitopes in the VD4 region. Particularly those containing the conserved neutralizing antibody epitope LNPTIAG were strongly and frequently recognized. The broad T cell response was unique to the Hirep1+CTH93/CAF01-immunized pigs and was not found in the UV-inactivated SvD-vaccinated group. This response was boosted following infection [35]. T cell epitopes in VDs have also been observed for infected humans, generating serovar-specific T cell responses [65].

### 4.2. Antigen Delivery Systems and Adjuvants

An overview of the antigen delivery systems and adjuvants used in research with MOMP-derivative constructs as vaccine antigens can be found in Table 3. Early research with synthetic peptides of MOMP from *C. trachomatis* showed only modest immune responses and protection, where VD4 peptides were conjugated to other proteins like *Schistosoma japonicum* glutathione S-transferase [78] and keyhole limpet hemocyanin [79]. The need for effective delivery systems for protein vaccines was clear, leading to extensive studies on vectors delivering hybrid constructs containing MOMP epitopes. These vectors are readily manufactured, easily manipulated, and are cost-effective [27]. Initially, attenuated pathogens were assessed since they can induce immunity against heterologous antigens and promote protection against the pathogen itself. Examples for MOMP multi-epitope constructs are the aroA mutant of *Salmonella typhimurium* [81], poliovirus type I Mahoney [83], cold-adapted influenza A/PR8/34 virus [75], Human Adenovirus serotype 5 and Modified vaccinia Ankara MVA pox [34]. Later on, non-pathogenic food-grade bacteria were considered a safer alternative for delivering MOMP-derived constructs [27], along with virus-like particles (VLPs). VLPs lack genetic material but self-assemble into particles due to their structural proteins. Examples include surface antigens of the Hepatitis B virus [52,72,73], the coat protein of the MS2 bacteriophage [58], and HPV major capsid protein L1 [59,60] or L2 [61]. These HPV VLPs that deliver a MOMP multi-epitope could potentially protect humans against both *C. trachomatis* and HPV infections, which is particularly interesting given the correlation between *C. trachomatis* and HPV infections [61].

A rather novel approach is the encapsulation of antigens within polymeric nanoparticles, which facilitates the controlled release of antigens depending on the matrix degradation rate. This allows for better recognition and uptake by antigen-presenting cells (APCs). These nanoparticles are safe, flexible in size manipulations, and capable of targeted or non-targeted delivery [96]. PLA-PEG nanoparticles have been studied in *C. trachomatis* vaccine research as they are comparable in size and shape to EBs and follow the same caveolin-endocytic pathway for their uptake and internalization by host cells, thus mimicking the natural chlamydial infection [85,87]. The CAF01 adjuvant is designed based on PLGA nanoparticles modified with the cationic surfactant DDA bromide and the immunopotentiator TDB. DDA binds antigens and targets the cell membrane of APCs, enhancing antigen uptake and presentation. TDB is a synthetic analogue of TDM, found in the mycobacterial cell wall [40]. The Th1/Th17 profile of CAF01 is well-suited for use as a *C. trachomatis* vaccine, explaining its frequent use in trials with MOMP-derivative antigen constructs [23,24,27,28,34,35,36,38,39,88,94,95]. CAF01 has been tested in phase I trials for HIV-1 and *Mycobacterium tuberculosis* infections, demonstrating an excellent safety profile in humans [97,98]. It has also passed clinical safety evaluations in phase I testing with CTH522 as the vaccine antigen, proving superior in inducing neutralizing serum antibodies and Th1 CMI responses compared to AlOH [99]. However, CAF01 is not optimal as a mucosal adjuvant [28]. Efforts to improve the mucosal adjuvant properties of PLGA/DDA/TDB nanoparticles by coating it with the mucoadhesive polymer glycol chitosan resulted in moderately higher anti-CTH522 IgG and IgA responses and similar systemic T cell responses [40]. It should be noted that mRNA vaccine platforms have not yet been investigated for MOMP-derived vaccine antigen constructs (Table 3).

### 4.3. Immunization Routes

Early research involved systemic vaccination with MOMP-derivative constructs, and observations revealed the need to explore other routes that could better stimulate immunity at mucosal sites [90,93]. Table 4 presents the immunization routes investigated for MOMP multi-epitope vaccines. Heterologous prime-boost strategies, involving different routes of administration for the same antigen, have proven superior to homologous prime-boost strategies in several studies [100]. Administering a mucosal booster vaccine on top of systemic immunity is particularly effective for achieving mucosal immunity [101]. Simultaneous delivery of systemic and mucosal MOMP subunit vaccinations has also been examined, potentially reducing doctor visits [28,39,88].

Antigen targeting to the NALT is effective in inducing high levels of specific immune effectors in the genital mucosa, making it suitable for vaccine delivery against genital chlamydial infection [75,102]. This approach has indeed been intensively explored for subunit MOMP vaccines as well [23,24,27,28,34,40,75,82,88,92,94,95]. Additionally, sublingual administration could be a patient-friendly alternative as a mucosal route. However, developing delivery systems that can protect antigens from salivary enzyme degradation and promote extended contact between antigens and the sublingual mucosa is challenging, often leading to poor antigen delivery due to swallowing [36]. Oral vaccination holds potential due to its low administration and logistics cost, as well as high patient compliance. However, subunit vaccines are highly sensitive to chemical and enzymatic degradation, necessitating innovative delivery systems and potent adjuvants [38]. Initial attempts at oral delivery of MOMP-derivative constructs were rather disappointing [81,89]. CTH522 formulated with α-GalCer, loaded into microcontainers with EL100-55 coatings, has been shown to be an efficient combination for oral vaccination [38].

## 5. Challenges for the Future

It is not possible to monitor all aspects of the immune response during each vaccine trial, because of the incredibly complex interaction between *Chlamydia* and the host [2]. Correlates of protection against *C. trachomatis* infection and reinfection have been associated with Th1 responses and IFN-γ secretion, whereas the role of antibodies is still debated [95]. The primary objective of a *C. trachomatis* vaccine will be to provide protection against long-term sequelae. Achieving sterilizing immunity will be highly unlikely [16]. However, biomarkers for ascension of infection and disease in the female upper genital tract are less validated [13]. For vaccine development to progress, clear clinical endpoints and specific biomarkers must be established [21]. It is also crucial to assess whether *C. trachomatis*’s immune evasion mechanisms could affect vaccine-induced immunity. [11]. Research into the best methods of immunization, vaccine antigens, adjuvants and delivery methods remains essential for developing an effective vaccine [16].

### 5.1. Broadly Protecting C. trachomatis Vaccine

An ideal *C. trachomatis* vaccine should include epitopes necessary to elicit a protective response against the majority of, if not all, serovars. Moreover, it should lend protection to hosts of different genetic backgrounds [90]. A possible pitfall for research regarding the peptide constructs of MOMP is the incorporation of a restricted number of T cell epitopes, which may compromise their immunogenicity in the general population due to HLA class I and II diversity [103].

A vaccine with low serovar coverage could lead to serovar emergence or replacement [88]. Immunity against all urogenital serovars by MOMP-derived vaccines has proven challenging as protective epitopes tend to be variable [58]. It has been hypothesized that a polyvalent vaccine based on the senior serovar of each complex would protect against all individual serovars [104]. In any case, it would be necessary for a vaccine to prevent infection of the GT by SvD, SvE, SvF and SvG [80], as these represent up to 90% of the genital *C. trachomatis* prevalence [23]. Promising are the multivalent Hirep constructs from the Statens Serum Institut, based on the extended VD4 regions from SvD, SvE, SvF and SvG, which promoted a strong antibody response that both amplified the response to the LNPTIAG region shared by the inserts and also increased the breadth of the response by including several serovar-specific VD4 antibody epitopes [23]. Such a strong cross-neutralizing antibody response, combined with responses to conserved T cell epitopes, could be the key for a *C. trachomatis* vaccine [88]. The CTH522 vaccine that underwent clinical phase I testing combines a large segment of MOMP from SvD with a Hirep construct (MOMP^D^_34–259_-extVD4^D^-extVD4^E^-extVD4^F^-extVD4^G^), as this segment contains shared B cell and T cell epitopes [99]. A possible scenario would be to supplement CTH522 with another repeat based on the VD1 regions from C-complex serovars to further broaden the serovar coverage [95]. This vaccine antigen could potentially serve a dual purpose as both an STI and trachoma vaccine [88]. A second phase I trial with the CTH522 antigen was set up. In addition to testing another cationic liposomal adjuvant, CAF09b, the ophthalmic immunization route is investigated. Results have not yet been published [105]. Consensus MOMP antigens have also been studied in the context of broad serovar coverage [34,106].

The MOMP locus is one of the most variable sites observed in whole-genome analyses, therefore significantly contributing to the occurrence of serovar-specific protection [11]. This large genetic diversity could be a possible pitfall in using MOMP as a single vaccine antigen, and the development of a multi-antigenic vaccine should be considered [2]. Polymorphic membrane proteins (Pmps) have been suggested as alternative candidates, as these surface-exposed proteins have highly conserved regions [1,2]. Pal et al. (2017) compared nine Pmps from *C. trachomatis* as vaccines in mice and although some gave immune responses and decreased signs of disease, MOMP was more effective [107]. A combination of the recombinant proteins PmpE, PmpF, PmpG, PmpH and MOMP accelerated clearance in mice [108]. Also, combination vaccines with, for example, chlamydial heat shock protein (cHSP)-65, outer membrane protein (OMP)2, type III secretion system proteins and inclusion membrane proteins have been studied, some offering protective responses [109,110,111,112]. The plasmid glycoprotein 3 (Pgp3), which is secreted into the cytosol of *Chlamydia*-infected cells [113], had an antagonistic effect on the ability of MOMP to elicit protection against a *C. muridarum* respiratory challenge when combined as a vaccine. This could be due to interference with MOMP-specific immune responses [114]. To our knowledge, MOMP-derived antigen constructs have only been combined with CT043 and CT414 (AA_605–804_) in a vaccination trial [28,35]. In addition to the antigens mentioned above, other targets could be interesting to combine with MOMP or MOMP-derived constructs. Examples include the chlamydial protease-like activity factor (CPAF), the type III secretion effector protein Tarp, the macrophage infectivity potentiator (MIP), CT584, the porin protein PorB, the ribonucleoside reductase NrdB, the glycolipid antigen-peptide 4 and glycogen phosphorylase [16].

### 5.2. Appropriate Animal Model

One major difficulty in the development of a *C. trachomatis* vaccine has been the quest for an appropriate animal model [21]. There has been a strong bias toward studies targeting *C. muridarum* and *C. trachomatis* within mice [2], which can be observed for vaccination trials with MOMP-derivative constructs as well (Table 5). A genital *C. muridarum* infection in mice is generally cleared within 4 to 5 weeks, and most mice develop ascending infection. In humans, on the other hand, *C. trachomatis* infects the GT for months to years and only 15% of women develop clinically apparent ascending infections [11], calling into question the relevance of the murine *C. muridarum* model. It should be noted that the dose of *C. muridarum* for challenge modulates the ascending infection [115]. However, one could argue that protection against ascending infections is especially needed, so that efficacy studies should be performed with the murine *C. muridarum* model, as *C. trachomatis* infections do not transmit ascending infections in mice either [93].

*C. muridarum* and *C. trachomatis* share considerable homology for MOMP. The conserved neutralizing LNPTIAG epitope that is the subject of many vaccine trials, however, is lacking in MOMP from *C. muridarum* [23,93]. Surprisingly, a decrease in *C. muridarum* burden was observed after vaccinating mice with the TTLNPTIAG epitope, expressed on VLPs [58]. Intravaginal administration of different *C. trachomatis* serovars in mice gave rise to infections with variable durations. The longest durations were observed for SvD and SvE, which could contribute to their high prevalences [116]. IFN-γ-mediated effector mechanisms leave *C. trachomatis* more vulnerable in the murine model, which means both vaccine-induced T cell and antibody effector mechanisms could be overestimated [95]. The non-ascending nature of *C. trachomatis* in mice can be overcome by transcervical or intrauterine challenge methods [21,93]. Pathology as a readout in murine models with human strains for challenge has, however, been the subject of discussion, as it is a concern that *C. trachomatis* infections might be eliminated predominantly by innate immune responses [23].

An important limitation for many studies is the use of inbred mice strains. Only six out of eight H-2 congenic mouse strains immunized with the A8-VD4 peptide produced high-titer IgG antibodies reactive with the VD4 peptide and full MOMP. Moreover, only two out of eight strains of mice produced IgG reactive with VD4 peptide and MOMP when immunized with the VD4 peptide alone [80]. Antigen delivery systems and adjuvants can play significant roles herein. A chimeric peptide comprising VD4 from SvE and VD1 from SvC induced little to no antibody responses in DBA/1 (H-2^q^) and BALB/c (H-2^d^) mice. However, when coupled to keyhole limpet hemocyanin (KLH), high antibody responses to both SvC and SvE, with in vitro neutralizing activity, were observed. It was suggested that KLH provides the necessary T cell help for a good antibody response [90]. Mouse strains of different H-2 haplotypes also vary dramatically in their responses to a genital challenge with *C. trachomatis.* Duration of infection, level of shedding and also quantitative and qualitative cellular and humoral responses differ significantly among mice strains [117]. For example, C57BL/6 (H-2^b^) mice are fairly resistant, with vaginal cultures being positive for only 1 to 2 weeks, depending on the challenge dose. C3H (H-2^k^) mice on the other hand are culture positive for 4 to 6 weeks when administered even low numbers of *C. trachomatis* [118].

The first phase I trial in humans with the CTH522 vaccine candidate [99] gave the opportunity to perform a comparative analysis between mouse and human immune responses regarding an MOMP-derivative construct as a vaccine antigen. Mapping showed that human responses are generally broader and more widely distributed throughout the MOMP sequence, compared to the inbred mouse responses after CTH522/CAF01 immunizations. In vitro stimulation of human PBMCs led to IL-17A, IFN-γ, IL-6, IL-13 and TNF-α as dominating cytokines, which resembles the cytokine profile in mice. Antibody responses were characterized by mouse IgG1, IgG2a/c and IgG2b, and human IgG1 and IgG3 subclasses. Serum antibodies from both mice and humans could neutralize SvD, SvE, SvF and SvG in vitro. Neutralizing epitopes were located in the VD4 region. Therefore, CTH522-specific immune responses in mice were translatable to humans to a high degree [95].

### 5.3. Long-Term Protection

In general, observations of long-lasting immunity (over 1 year) are lacking in chlamydial vaccine research [2]. However, a *C. trachomatis* vaccine will need to induce long-lived protection, at least covering the age group of 15 to 29 years, where the infection is most prominent [94]. A future vaccine is likely to be introduced in parallel with the HPV vaccine, thus in adolescents before their sexual debut, and therefore protection needs to be sustained for at least a decade [95]. The focus of future studies should lie on defining those parameters that govern long-term protective immunity [37]. Over time, the role of neutralizing antibodies in the immediate control of infection will likely decline, and instead, immunity will rely on the memory T and B cell response [95]. Long-term protective immunity has correlated with the preservation of a robustly high frequency of specific Th1 cells and elevated IgG2a levels in genital secretions when mice were immunized with a chlamydial-pulsed IL-10-deficient DC-based cellular vaccine, or with live-attenuated influenza viruses as delivery vectors for T cell epitopes of MOMP from *C. trachomatis* in a murine genital infection model [75,119]. The common immune signature between mice and humans vaccinated with CTH522 was found to not only protect mice against ascending infection and pathology, but also proved to sustain protection for up to 1 year. These observations have led to the proposal for further investigation of CTH522 in a phase IIb study [95].

## 6. Conclusions

Urogenital infections with *C. trachomatis* serovars D–K are a global health concern, making vaccination research a priority. Research in mice indicates that a successful *C. trachomatis* vaccine should elicit both humoral and cell-mediated immune responses, including mucosal IgG and IgA with neutralizing capabilities, and Th1 responses involving IFN-γ. Mucosal immunization has gained attention in *C. trachomatis* vaccination. While effective in generating local immunity (TRM T and B cells, and sIgA), mucosal immunization often requires preceding systemic vaccination for optimal efficacy. Of interest is the effective interaction between certain sites of the common mucosal immune system. However, some studies suggest that parenteral vaccination alone may be a viable strategy for *C. trachomatis* protection.

MOMP has emerged as the most promising vaccine antigen candidate. Extraction of nMOMP from *Chlamydia* is impractical. Efforts to produce functional rMOMP protein for vaccine development are hindered by challenges such as protein misfolding, poor solubility, and low yield. To overcome these problems, studies have emerged on MOMP-derived vaccine antigens, which can still benefit from the antigenic properties of MOMP. The significance of the VD4 region of MOMP in neutralizing antibody responses was recognized early. Particularly, the VD4 region and its surrounding membrane anchor have shown promising results. The inclusion of T cell epitopes, in the form of universal T cell helper peptides, other MOMP regions, or (regions of) other *C. trachomatis* antigens, has improved immunogenicity of the MOMP-derived constructs. However, these constructs often require an antigen delivery system or accompanying adjuvant to generate adequate co-stimulation and a favorable cytokine environment. Particularly, vectors delivering hybrid constructs, VLPs and polymeric nanoparticles have been studied for MOMP-derived constructs. These constructs have proven to be promising vaccine candidates in systemic, mucosal and combined vaccine strategies. The CTH522 candidate has completed its first clinical phase I testing and is currently in a second phase I testing. Attention to long-term, broad-serovar coverage protection, and the shortcomings of specific animal models is necessary to accelerate the creation of a vaccine. Continued and diversified research to overcome the existing challenges in *C. trachomatis* vaccine development is critically needed.

## Figures and Tables

**Table 1:** A general comparison of vaccination strategies in terms of efficacy, safety, mechanism of action, cost and accessibility [41].

Vaccine Type	Efficacy	Safety	Mechanism of Action	Cost	Accessibility
Live attenuated vaccine	High, often strong and long-lasting immunity	Risk of reversion, not suitable for immunocompromised individuals	Mimics natural infection (humoral and cellular responses)	High, cultivation and ensuring vaccine remains attenuated	Cold chain storage, careful handling
Inactivated vaccine	Moderate, may require boosters and/or adjuvants	Considered safe	Mainly humoral immune responses	Moderate	Cold chain storage
Subunit vaccine (protein based, vector vaccines, …)	Moderate to high, with right antigen and delivery system/adjuvant combination	Considered safe	Targeted immune response, depending on platform	Variable, depending on platform	Often requires cold chain storage
DNA vaccine	Moderate, optimization necessary	Considered safe (low risk of integration in genome)	Targeted humoral and cellular response	Low to moderate, scalable	Stable at room temperature
RNA vaccine	High	Considered safe	Targeted humoral and cellular response	Low to moderate, scalable	Ultra-cold storage, ongoing research to improve stability

**Table 2:** Overview of evaluated MOMP-derived constructs, along with the observed immune responses and protective efficacy outcomes.

MOMP-Derived Antigen Construct	Immune Responses	Protective Efficacy
Non-chimeric MOMP fragment		
VD4 (complete/partial)	Serum antibody response [58,78,79,80,81,82]—low local antibody response, T cell response [58,82]	In vitro neutralization [78,79]—reduced colonization [58]
VD1	Serum antibody response [83]	In vitro and passive in vivo neutralization [83]
TINK	No antibody response, T cell response [84]	Reduced salpingitis [84]
MOMP_370–387_	Serum and local antibody response, cytotoxic T cell response, IFN-γ and IL-4 [73]	Reduced colonization and histopathology [73]
MOMP278	Serum and local antibody response [85,86,87]—IFN-γ [85,87]	Reduced in vitro infectivity macrophages [85]—in vitro neutralization and reduced colonization [87]
ExtVD4	Serum antibody response [23]	In vitro neutralization [23]
ExtVD1	Serum and local antibody response, IFN-γ [88]	In vitro and passive in vivo neutralization, reduced colonization and enhanced clearance [88]
¾ or ½ rMOMP	Serum antibody response [89]	Enhanced clearance and reduced salpingitis [89]
Chimeric MOMP fragments		
VD4-VD1	Serum antibody response [90,91]—T cell response [90]	In vitro neutralization [90]—no in vitro neutralization [91]
VD1-VD4	Serum antibody response [91]	In vitro neutralization [91]
VD2-VD4	Serum and local antibody response, IFN-γ, IL-13 and IL-17 [92]	Reduced shedding, better protection against infertility [92]
A8-VD4	Serum antibody response [80,93]—local antibody response [93]	In vitro neutralization, no protection in NHPs [80]—no significant reduction in colonization, shedding or enhanced clearance [93]
Hirep1/2 ExtVD4*4	Serum antibody response [23,27,94]—low local antibody response [27]—Th1/Th17 response [23]—IFN-γ [27]—broad CMI response [94]	In vitro and passive in vivo neutralization [23,94]—reduced colonization and pathology [23]—short- and long-term protection [94]
TINK + P12	Th1 response [75]	Reduced shedding and enhanced clearance [75]
MOMP168	Serum and local antibody response [42,52,59,61]—IFN-γ [42,59]—cytotoxic T cell response [42,52,61]	Reduced shedding and enhanced clearance [42,52,59,61]—no salpingitis [52]—no histopathology [61]
MOMP_370–387+261–276+70–81_	Serum and local antibody response, cytotoxic T cell response, IFN-γ and IL-4 [72]	Reduced shedding and oviduct pathology, enhanced clearance [72]
CTH522	Serum antibody response [23,28,34,36,38,39,40,95]—local antibody response [34,36,38,39,95]—IFN-γ [28,40]—IL-17 [28,36,38,95]—Tissue-resident Th1/Th17 response [39]—broad CD4^+^ (and CD8^+^) T cell response [34,95]	In vitro neutralization [23,34] and passive in vivo neutralization [95]—reduced colonization [23,39]—enhanced clearance [34]—long-term protection against ascending infection and pathology [95]
Combinatorial approach		
Hirep + CTH93	Serum and local antibody response, IFN-γ [24,28,35]—IL17A [24,28]	In vitro neutralization [24]—enhanced clearance [24,28]

**Table 3:** Overview of antigen delivery systems and adjuvants evaluated for MOMP-derivative vaccine antigens. The term fusion protein refers to the coupling of, or the integration into, a foreign protein.

Vaccine Type	Delivery System	(Extra) Adjuvant	References
Protein vaccine	None	None	[34,73,84,86,88]
		Hydrogel suspension	[34,36,89,93]
		Oil-in-water emulsion	[80,90,91]
		Toxin	[92]
	Fusion protein	Oil-in-water emulsion	[42,78,79,90,91]
	Vector	None	[34]
	Fusion protein + Vector	None	[27,75,81]
		Oil-in-water emulsion	[83]
	Nanoparticles	None	[85,87]
		On surface nanoparticles	[23,24,27,28,34,35,36,38,39,40,82,88,94,95]
	Fusion protein + Nanoparticles	None	[58,72,73]
DNA vaccine	Plasmid	None	[34,60,61]
	Plasmid + Fusion protein + Nanoparticles	None	[52,59,60,61]

**Table 4:** Overview of immunization routes evaluated in animal trials with MOMP-derivative vaccine antigens.

Immunization Route		References
Systemic	Intramuscular	[24,34,35,52,58,59,60,61,78,80,82,83,86,89]
	Subcutaneous	[23,27,28,36,38,39,42,72,73,78,83,85,87,88,89,93,94,95]
	Intravenous	[78,81]
	Intraperitoneal	[79,80,89,90,91]
	Intradermal	[34,84]
	Presacral	[82,89]
Mucosal	Oral	[38,81,89]
	Intranasal	[23,24,27,28,34,40,75,82,88,92,94,95]
	Intravaginal	[89,92]
	Intrauterine	[39]
	Sublingual	[36]
	Peyer’s patch	[89]

**Table 5:** Overview of animal models in vaccination trials with MOMP-derivative vaccine antigens.

Animal Model		References
Mouse	One strain	[27,28,36,38,39,40,42,52,58,59,60,61,72,73,75,79,81,85,86,87,89,91,93,94,109]
	Multiple strains	[23,80,82,84,88,90,92,95]
Rabbit		[78,83]
NHP		[34,80]
Minipig		[24,35]

## Data Availability

No new data were created.

## Supplementary Materials

The following supporting information can be downloaded at https://www.mdpi.com/article/10.3390/microorganisms12061196/s1, Table S1: Overview of MOMP-derived constructs tested as vaccine antigens in animal models over the years.
