# Supplementary material for: Advances in Chlamydia trachomatis Vaccination: Unveiling the Potential of Major Outer Membrane Protein Derivative Constructs
Source: Microorganisms. 2024 Jun 13;12(6):1196. doi: 10.3390/microorganisms12061196 (PMC11205628; doi:10.3390/microorganisms12061196)
Supplement: Supplementary file 1 [file microorganisms-12-01196-s001.zip › microorganisms-3006669-supplementary.pdf]

**Authors**<br>**Year**<br>**VACCINE TYPE**	**Epitope information**	**Delivery system/adjuvant**	**Animal model + immunization set-up**	**Results**	**Remarks**
[1] Toye – Brunham<br>1990<br><br>PROTEIN VACCINE - CONJUGATE	183bp fragment encoding VD4 ($MOMP_{273-333}$) of SvB	Linked to *Schistosoma japonicum* glutathione S-transferase (GST)<br><br>Complete & incomplete Freund's adjuvant	New Zealand White rabbits i.m. 1.2 mg + complete Freund (d0); s.c. 500 µg + incomplete Freund (d7,d14); i.v. 100 µg (d21)	Antisera recognized MOMP from all 12 tested serovars; EBs from B, D, E, L2 (B-complex) & K (strong); F, G, A, H (weak); C, J, I (not)<br>5 linear epitopes recognized; only 2 are surface exposed on serovar B EB (SAETIFD, IFDVTTL). Latter also on D, E, F, L2 & K.<br>Epitope TLNPTIA surface exposed on A, D, E, F, K, L2 & L3.<br>Neutralizing serovars B, D, K, L2, F, G; not J	Epitope 1, located within constant region upstream from VD4 not found when whole EBs were used to raise antisera; region not surface exposed on serovar B EBs
[2] Hayes – Clarke<br>1991<br><br>PROTEIN VACCINE - CONJUGATE + LIVE ATTENUTATED VECTOR	- Epitope B1 $MOMP_{288-298}$ SvB SAETIFDVTTL (in VD4)<br>- Epitope B2 $MOMP_{298-308}$ SvB LNPTIAGAGDV (in VD4)	B1+B2 fused to LamB protein (porin of *E. coli*) in outer membrane of *aroA* mutant of *Salmonella typhimurium* (colonizes mouse gut, but doesn't multiply)	C3H/He/Ola mice (6-10 weeks)<br>i.v. with 0.1 ml of 2x10^6/ml or orally with 0.1 ml of 2x10^11/ml	Serum IgG responses to *S. typhimurium* and Ct were induced but only in fraction of the animals (more for oral imm). Neither mucosal response (gut washes) nor serum IgA was detected.	
[3] Cheng – Peterson<br>1992<br><br>PROTEIN VACCINE - CONJUGATE	CSATAIFDTTTLNPTIAG AGDVKASAEGQLG<br>30 amino acids of VD4 of SvE with additional N-terminal C	Peptide linked to keyhole limpet hemocyanin (KLH)<br><br>Complete & incomplete Freund's adjuvant	BALB/c mice (4-6 weeks)<br>2x i.p. 100 µg KLH-VD4; 2-week interval	Resulting antisera recognized all 15 serovars of Ct<br>Two main regions recognized:<br>- N terminus; SATAIFD ; B-complex specific<br>- Middle region; TLNPTIA; species-conserved<br>Neutralizing mainly serovars in B complex (except L2); E and D to greatest extent (identical sequence N terminus VD4)	
[4] Tuffrey – Ward<br>1992<br><br>PROTEIN VACCINE	¾ or ½ length rMOMP (from C-terminus) of SvL1 (produced in *E. coli*)	Adsorbed to alhydrogel	C3H mice (6-8 weeks)<br>Exp 1) 20 µg ¾ rMOMP; d0, d14 & d21; s.c. + i.m. + i.p. (parenteral)/ into Peyer's patch / orally; challenge i.vag or i.u. with 5x10^4 IFUs Ct strain NI 1 on d28<br>Exp 2) 100 µg ¾ rMOMP; d0, d7, d14; into Peyer's patch + 200 µg ¾ rMOMP; d20-d22; i.vag (or i.vag imm alone); challenge i.u. with 5x10^4 IFUs Ct strain NI 1 on d30<br>Exp 3) 20 µg ½ rMOMP; d0, d14, d28; orally/ s.c. /	Parenteral imm with ¾ and ½ rMOMP reduced duration of colonization and severity of salpingitis, coincided with high levels of circulating IgG.<br>Insufficient to prevent infertility following direct inoculation into ovarian bursae.<br><br>Direct imm into Peyer's patches more effective at reducing colonization, despite absence of significant local Ab. Neither i.vag boosters, nor imm via presacral space achieved significant levels of Ab in GT secretions.	Fertility study? Challenge into ovarian bursae

			i.p. / presacral space (+ 50 µg ½ rMOMP; d32 & d44, i.vag); challenge i.u. with 5x10^4 IFUs Ct strain NI 1 on d53		
[5] Murdin – Caldwell 1993<br><br>PROTEIN VACCINE – CONJUGATE + LIVE VIRAL VECTOR	Poliovirus hybrid: part of neutralization antigenic site I (NagI) of poliovirus type I Mahoney (PV1-M) replaced by VD1 of SvA (TTSDVAGLEKDPVA included neutralization epitope VAGLEK)<br>= PV1-Ct7<br><br>Compared to:<br>PV1-XLD (wild-type)<br>MOMP (SvA)<br>Synthetic peptide A8-VD1 (SvA)	Complete & incomplete Freund's adjuvant	Rabbits<br>1st s.c. dose + i.m. dose; 2nd & 3rd s.c. dose; 2-week interval<br>Dose of +/- 3.0 x 10^11 virions, 1.2 µg MOMP, 0.16 µg peptide (all 30 pmol)	PV1-Ct7 antisera reacted strongly with SvA, moderately with SvC, not with SvB (MOMP and A8-VD1 antisera lower and more variable response)<br>Marked immunoreactivity with VD1 octapeptides containing VAGLEK; neutralizing activity in vitro for SvA (and SvC) and in vivo (passively neutralized SvA infectivity in conjunctival cells of cynomolgus monkeys, no clinical signs of conjunctivitis + culture negative)	Comparison of hybrid viral construct to purified SvA MOMP or A8-VD1 peptide alone (expected better mucosal immune response for hybrid vaccine)<br><br>Live poliovirus as vaccine (however doesn't replicate in rabbits)
[6] Su – Caldwell 1993<br><br>PROTEIN VACCINE	1) A8-VD4 peptide<br>A8 = $MOMP_{106-130}$ SvA ALNIWDRFDVFCTLGATTGYLKGNS; Th epitope + VD4 = $MOMP_{293-309}$ SvB FDVTTLNPTIAGAGDVK contains LNPTIAG; species-common neutralizing epitope<br>2) VD4 peptide	Mice: complete & incomplete Freund's adjuvant<br><br>Monkeys: Ribi adjuvant (MPL + TDM + CWS)	C57BL/10SnJ ($H-2^b$), B10.A/SgSnJ ($H-2^a$), B10.D2/oSnJ ($H-2^d$), B10.M/Sn ($H-2^f$), B10.WB/Sn ($H-2^{ja}$), B10.BR/SgSnJ ($H-2^k$), B10.PL(73NS)/Sn ($H-2^u$) and B10.SM(70NS)/Sn ($H-2^v$) mice (8-12 weeks)<br>2x i.p. 50 µg peptide; 3-weeks apart<br><br>Cynomolgus monkeys (*Macaca fasicularis*) of Mauritius NHP Island origin<br>i.m. 1 mg A8-VD4 peptide + 2x 500 µg; 4-week interval	6/8 mouse strains (b,a,f,ja,k,v) IgG responses when A8-VD4 imm; 2/8 strains (b,f) IgG responses when VD4 imm; only IgG responses to VD4 and whole MOMP (not A8)<br><br>5/6 strains: Ab with enhanced immunoreactivity to LNPTIAG epitope; Ab reacted with B complex Ebs (D and E) & intermediate complex (F, G & K), weak or not with C complex (H, I & J); neutralizing activity for D and G, not for H<br><br>All three NHP produced sig IgG, reactive to peptide VD4 and Ct SvD, E, F, G & K (not H, I & J); strong reactivity to LNPTIAG; neutralizing activity for D and G, not for H	Mice with certain H-2 haplotypes incapable of recognizing the Th epitope in A8/VD4<br><br>NHP challenged cervically with Ct SvD; no protection (reisolation from cervical specimens), suggested that neutralizing antibodies were not translocated to mucosa
[7] Qu – Peterson 1994<br><br>PROTEIN VACCINE - (CONJUGATE)	Chimeric peptide NH2-CSATAIFDTTTLNPTIAGAGDVKASACAAPT TSDVAGLQNDPTTNVA-COOH (VD4 SvE + VD1 SvC) containing neutralizing	Alone or coupled to keyhole limpet hemocyanin (KLH) (effective carrier to ensure necessary T-cell help for good antibody response)	A/J ($H-2^a$), DBA/1 ($H-2^q$), C57BL/10 ($H-2^b$), CBA/J ($H-2^k$) and Balb/c ($H-2^d$) mice (6-8 weeks)<br>3x i.p. 100 µg peptide (d0,d21,d35)	Ab responses differed through time for mice strains, at end trial: all strains except d Abs to SvE and SvC (titers varied greatly); antisera recognized all serovars in dot blot assay; strong reactivity to neutralizing epitopes; neutralizing activity to B complex (B,D,E), C	Little to no detectable chlamydial antibody in vaginal washes (data not shown)

	epitopes VAGLQNDPT in VD1 and TLNPTIA (species-conserved) in VD4	Complete & incomplete Freund's adjuvant	DBA/1 (H-2^q), C57BL/10 (H-2^b) & Balb/c (H-2^d) mice KLH-peptide or KLH alone<br><br>i.p. 100 µg peptide d0 + 1x10^6 IFUs SvC & SvE d21 for lymphocyte proliferation assay on splenocytes	complex (C,J), B-related (F) and C-related (L3) serovars<br><br>T cell proliferative response to chimeric peptide, SvC and SvE in splenocytes<br><br>All strains gave high titers to SvC and SvE when immunized with KLH-peptide; recognized neutralizing epitopes; neutralized SvC and SvE in vitro	
[8] Knight – Tuffrey 1995<br><br>PROTEIN VACCINE	TINKP peptide, 12 AA located on the edge of VD3	/	C3H/He-mg (H-2k) and BALB/c (H-2d) mice (6-8 weeks)<br>1x i.d. 10 µg TINKP/ 20 µg SvF MOMP/ 3.6x10^3 IFUs NI1<br>4x i.d. 10 µg TINKP (divided over 2 sites); one-week interval<br><br>Challenge: i.u. with 1.5x10^4 (C3H) or 5x10^4 (BALB/c) IFUs NI1 one week later	No antibody responses observed<br><br>Cell-mediated immunity since recall responses in T cells observed.<br><br>Some protection against salpingitis	Comparison with MOMP SvF and SvF EB.<br><br>TINKP generated primary proliferative T-cell responses in cells from 80% of naïve human volunteers, suggesting it's a promiscuous, non-haplotype-restricted peptide. Also conserved in all Ct serovars.
[9] Su – Caldwell 1995<br><br>PROTEIN VACCINE	A8-VD4 peptide<br>A8 = $MOMP_{106-130}$ SvA ALNIWDRFDVFCTLGATTGYLKGNS; Th epitope + VD4 = $MOMP_{293-309}$ SvB FDVTTLNPTIAGAGDVK contains LNPTIAG; species-common neutralizing epitope	Adsorbed with aluminum hydroxide gel (Rehydragel LV)	Mice A/J (8-12 weeks)<br>3x s.c. 100 µg A8-VD4 peptide + 0.6 mg alum; 30 day interval<br>Challenged i.vag or i.u. with 10, 10^2 or 10^3 ID_{50} / 3x10^2, 3x10^3 or 3x10^4 IFUs	High levels of anti-chlamydial serum IgG neutralizing antibodies, lower but detectable levels in vaginal washes (IgG1, not IgA)<br><br>No statistically significant differences in colonization, shedding or duration of infection but a trend indicating lower incidence of infection and recoverable IFUs for low and medium dose challenge groups (both i.vag and i.u. challenge)	
[10] Peterson – de la Maza 1996<br><br>PROTEIN VACCINE - (CONJUGATE)	Synthetic peptides of 27 AA representing portions of VD1 of SvC and VD4 of SvE (+ N-terminal cysteine)<br>VD1-4: CVAGLQNDPTSATAIFDTTTLNPTIAG<br>VD4-1: CSATAIFDTTTLNPTIAGVAGLQNDPT	Alone or coupled to keyhole limpet hemocyanin (KLH) (effective carrier to ensure necessary T-cell help for good antibody response)<br><br>Complete & incomplete Freund's adjuvant	C57BK/10 mice<br>2x 100 µg peptide/ KLH conjugate; i.p.; two-week interval	VD1-4 antisera gave peak antibody response in VD4 (and only to SvE); VD4-1 antisera gave peak antibody response in VD1 (and only to SvC). Thus carboxy terminus of chimeric peptide recognized by antisera.<br>Also Abs directed to epitopes unique to the chimera.<br><br>VD1-4 antisera but not VD4-1 antisera were able to neutralize SvE in vitro. No neutralization of SvC.	Assessment whether position within chimeric peptide influences immunogenicity of epitopes.

				Ab titers among KLH and non-KLH conjugated immunogens were similar.	
[11] Motin – Peterson 1999<br><br>PROTEIN VACCINE - NANOPARTICLE	VD4 peptide $MOMP_{309-338}$ SvE Co-administered with T-cell helper peptides from *universal<br>- Tetanus toxin $_{830-844}$*<br>- Pre-S2 region of hepatitis B virus $_{120-132}$*<br>- Mouse HSP60 $_{458-474}$*<br>- Ct HSP60 $_{435-451}$	Encapsulated in liposomes containing adjuvant monophosphoryl lipid A	C3H (H-2k) and C57BL/6 (H-2b) mice (6-7 weeks)<br>25 µg VD4 peptide (+ 25 µg T-cell helper peptide); primo i.m. d0; 2x booster both presacral and i.n. d21, d35	Ct HSP60 only T-cell helper peptide that improved immune response.<br><br>Serum Ab levels increased in C3H mice (weak responders to VD4 alone), not significantly enhanced in C57BL/6 mice (good responders to VD4 alone). Ab levels both strains are similar, however, pepscan profile and Ig subclass profile were different and neutralizing titers and T-cell proliferation responses still lower for C3H mice. T-cell helper responses different for C3H mice (in Ct HSP60) than for C57BL/5 mice (in VD4).<br><br>In both strains titers of vaginal antibody to Ct were low.	
[12] He – Igietseme 2007<br><br>PROTEIN VACCINE – CONJUGATE + LIVE ATTENUATED VIRAL VECTOR	Immunodominant T-cell epitopes into stalk region of neuraminidase (NA) gene:<br>- TINK epitope $MOMP_{232-243}$ SvD TINKPKGYTGKE = PR8-TINK<br>- P12 epitope $MOMP_{293-304}$ SvD SFDADTIRIAQP = PR8-P12	Live attenuated cold-adapted influenza A/PR8/34 virus (H1N1) (FDA-approved)	C57BL/6 mice (5-8 weeks)<br>2x i.n.; 20 µl with 10^5 PFUs of PR8-TINK / PR8-TINK + PR8-P12; two-week interval<br>Challenge: i.vag with 10^5 IFUs Ct SvD in 20 µl PBS<br>Reinfection 97 days after infection.	Strong Th1 responses against Ct EBs. High frequency of Ct-specific Th1 in genital mucosal and systemic draining lymphoid tissues within 24 hr after challenge.<br><br>Protective immunity by shedding less and rapid clearing. Long-term immunity correlated with robust high frequency of Th1 cells and IgG2a in genital secretions.	Multiple epitope constructs provided a vaccine advantage over single recombinants.<br><br>Purified immune T cells (> 96% CD4/CD4) adoptively transferred from vaccinated to naïve mice, challenged after 24h.
[13] Yang – Wang 2007<br><br>PROTEIN VACCINE	Fusion protein Hsp65-MOMP-T-epitopes (H-ctm1)	/	C57BL/6 mice (6-8 weeks)<br>Challenged with Ct i.vag	Significantly lower Ct load in vaginal tract for H-ctm1 than HK-EBs group (d9 and d18 pi). Inflammation scores significantly lower in H-ctm1 and HK-EBs groups.<br><br>Unilateral or bilateral hydrosalpinx present is all control animals, not found in immunized animals.	Comparison to heat-killed EBs (HK-EBs)<br><br>No details since no open access
[14] Shi – Zhang 2010<br><br>DNA VACCINE – (CONJUGATE + NANOPARTICLE)	Ct MOMP multi-epitope<br><br>Alone or connected to C-terminal end of HPV type 6b capsid protein L1	pcDNA3.1(+)-HPV6b L1-Ct MOMP / pcDNA3.1(+)-Ct MOMP / pcDNA3.1(+)	BALB/c mice<br>3x i.m.; 150 µg plasmid; 2-week interval	Significant Ct MOMP multi-epitope specific cytotoxicity of CTL in spleen by both constructs, significantly higher for chimeric construct (also HPV6b L1 specific cytotoxicity of CTL in spleen).<br><br>Significantly higher intracellular IFN-γ level in CD3+ T cells in spleen by chimeric plasmid construct. No differences in IL-4 and IL-10 levels.	No details since no open access.

[15] Taha – Dennis 2011<br><br>PROTEIN VACCINE	$MOMP_{278\text{-}370}$ = rMOMP-278 (fragment containing T-cell epitopes)	/	BALB/c mice (4 to 6 weeks)<br>3x i.m.; 50 µg rMOMP / rMOMP-278	Total antibody responses higher for rMOMP immunized animals than for rMOMP-278 immunized mice (both in serum and vaginal washes).<br>Isotypes: IgG2b > IgG2a > IgG1 for rMOMP-278 (mixed Th1/Th2 response); IgG1 > IgG2b > IgG2a for rMOMP (dominant Th2 response). IgA levels in serum and vaginal washes similar for both groups.	Comparison to rMOMP.
[16] Xu – Zhang 2011<br><br>DNA VACCINE – CONJUGATE + NANOPARTICLE	SYFPEITHI program<br>GDNENQSTVKTNSVP<br>NMSLDQSVVELYTWQ<br>ASLALSYRLNMFTPYIGV<br><br>SvE $MOMP_{138\text{-}165}$ + $MOMP_{249\text{-}268}$ ("MOMP 168")<br>Th, HLA-A2 CTL, B-cell	pcDNA3.1(+)/HPV6bL1/Ct MOMP168<br><br>Human papillomavirus (HPV) major capsid protein L1, forms virus like particles (VLPs) in eukaryotic cells; capable of inducing strong humoral and cellular IR in mucosal tissues	BALB/c mice (6-8 weeks)<br>5x i.m. with 200 µg; two-week interval<br>Challenge: 3 weeks after final immunization, i.vag, 10^7 IFUs Ct SvE	IFN-γ production by splenic T cells, no IL-4<br>CTL lysis sig. greater<br>Sig specific IgG antibodies in serum – highest titer after 8 weeks; at 6 weeks sig IgA levels in the genital mucosa<br>highly resistant to infection (low IFU and time to resolution)	Chimeric vaccine has immunologic advantage over the single unit construct (pcDNA3.1(+)/Ct MOMP168)
[17] Zhu – Zhang 2014<br><br>DNA VACCINE – CONJUGATE + NANOPARTICLE	SYFPEITHI program<br>GDNENQSTVKTNSVP<br>NMSLDQSVVELYTWQ<br>ASLALSYRLNMFTPYIGV<br><br>SvE $MOMP_{138\text{-}165}$ + $MOMP_{249\text{-}268}$ ("MOMP 168")<br>First part: H2-Kd CTL<br>Both parts: Th, HLA-A*0201 CTL, B-cell<br>Well conserved (D & E)	pcDNA3.1(+)-HBsAg-MOMP168 / pcDNA3.1(+)-MOMP168-HBsAg<br><br>Safe (vaccine against HBV); Numerous studies that HBsAg tolerates insertion of foreign sequences and induce specific immune responses against the foreign antigens	BALB/c (H2-Kd) mice<br>3x i.m. with 100 µg; two-week interval<br>Challenge: 3 weeks after 3rd immunization, i.vag, 10^5 IFUs of Ct SvE in 20 µL SPG	Sig increased cytotoxicity of splenocytes<br>Sig increased Ct-specific IgG in serum and sIgA in vaginal secretions<br><br>Shorter resolution time; for best performing group 15 days (30 days for control) + sig reduction in titers in vaginal swabs + normal oviduct lumens	Group that received pcDNA3.1(+)-HBsAg-MOMP168 was better performer
[18] Dixit – Dennis 2014<br><br>PROTEIN VACCINE - NANOPARTICLE	M278 peptide = $MOMP_{278\text{-}370}$ (fragment containing T-cell epitopes)	Encapsulated in PLA-PEG nanoparticles (self-adjuvanting)	BALB/c mice (6-8 weeks)<br>3x s.c. 50 µg PLA-PEG-M278 / M278 peptide; 2-week interval	Higher T cell cytokine (IFN-γ, IL-2, IL-17) and serum antibody (IgG2a, IgG1 and IgG2b) responses compared to bare M278. Serum inhibited Ct infectivity of mouse J774 macrophages (reduction in MOMP, TLR2 and CD80 expression in macrophages).	
[19] Tu – Zhang 2014<br><br>PROTEIN VACCINE – CONJUGATE	SYFPEITHI program<br>$MOMP_{160\text{-}187}$ SvE (GDNENQSTVKTNSVPNMSLD QSVVELYT) +<br>$MOMP_{271\text{-}290}$ SvE (SvD) (WQASLALSYRLNMFTPYIGV)<br><br>Fusion protein Trx-His-MOMP multi-epitope	Complete & incomplete Freund's adjuvant	BALB/c mice<br>3x s.c. 100 µg; 2-week interval<br>Challenge i.vag with 10^6 IFUs Ct in 20 µl SPG, d14 after last vaccination	Strong IgG1 and IgG2a responses (mixed Th1/Th2 response with bias to Th2).<br>Moderate IgA response in vaginal lavage fluid.<br><br>Robust cytotoxic T lymphocyte response.<br>Elevated IFN-γ in serum (not IL-4).<br><br>Partial protection: reduction of Ct shedding and accelerated clearance.	The MOMP multi-epitope of 48 AA comprises at least 7 overlapping human HLA-A2-restricted CTL epitopes, 2 mouse H2-Kd-restricted CTL epitopes, 1 Th epitope and 1 B-cell epitope.

[20] Jiang – Zhang 2015<br><br>PROTEIN VACCINE – (CONJUGATE – NANOPARTICLE)	SYFPEITHI program: MOMP$_{370-387}$ SvE (contains HLA and H2 restricted CTL/Th epitopes and reported TRLIDERAAH B cell epitope)<br>Hepatitis B virus core antigen (HBcAg) can self-assemble into virus-like particles (VLPs)<br>- MOMP$_{370-387}$ alone<br>- HBcAg – MOMP$_{370-387}$<br>- HBcAg – Th – MOMP$_{370-387}$<br>- Th – MOMP$_{370-387}$ – HBcAg<br>- Th – HBcAg(N) – MOMP$_{370-387}$ – HBcAg(C) (inserted into MIR)<br>With Th universal PADRE epitope	HBcAg - VLPs	BALB/c mice (6-8 weeks)<br>3x s.c.; 100 µg; 2-week interval<br>Challenge: i.vag with 10^6 IFUs Ct SvE at week 8	All fusion construct elicited immune responses (IFN-γ and IL-4 levels in serum, IgG and sIgA responses, CTL activity in splenic lymphocytes) and protection to some degree (vaginal load and histopathology). Generally better responses than MOMP$_{370-387}$ peptide, and lower or similar responses than inactivated EBs.<br><br>The N-terminus fusion (Th – MOMP$_{370-387}$ – HBcAg) exhibited best immunogenicity and protection.<br><br>Universal Th1 epitope enhanced immunogenicity.	Comparison to inactivated EBs (similar cytokine levels in serum, highest IgG and sIgA antibody levels, highest CTL activity in spleen, best protection)
[21] Lorenzen – Andersen 2015<br><br>PROTEIN VACCINE - NANOPARTICLE	Hirep1 = extVD4^{D}-extVD4^{E}-extVD4^{F}<br>CTH93 = CT043 – CT414$_{605-804}$ – MOMP$^{D}_{34-371}$	Emulsified in cationic adjuvant formulation 1 (CAF01)<br><br>(+ 9% sucrose, isotonic solution)	SPF sexually mature female Göttingen minipigs (5-6 months)<br>2x i.m. 5 µg Hirep1 + 5 µg CTH93; adjuvanted; d0 & d14 (all groups)<br>2x i.n. 100 µg Hirep1 + 100 µg CTH93; adjuvanted or unadjuvanted; d42 & d56 (2 groups)<br>Challenge: vaginocervically with 5x10^9 IFUs Ct SvD in SPG on d85	IM priming induced significant cell-mediated IFN-γ and IL-17A response and significant systemic high-titered neutralizing IgG response.<br><br>IN boosted groups mounted accelerated, highly significant genital IgA response post challenge, that correlated with enhanced clearance on d3 pi. Genital IgA was locally produced.	Evaluation of intramuscular prime/intranasal boost vaccination strategy for induction of local genital immunity.<br><br>Confirmation that nasal-genital bridge exists in pigs.
[22] Olsen – Andersen 2015<br><br>PROTEIN VACCINE - NANOPARTICLE	VD4epitope = MOMP^{E}292-308 (containing LNPTIAG)<br>VD4^{E} = MOMP^{E}287-316<br>VD4^{F} = MOMP^{F}288-318<br>ExtVD4^{E} = MOMP^{E}260-327<br>ExtVD4^{F} = MOMP^{F}261-329<br>Hirep1 = extVD4^{D}-extVD4^{E}-extVD4^{F}<br>Hirep2 = extVD4^{D}-extVD4^{E}-extVD4^{F}-extVD4^{G}<br>CTH522 = MOMP^{D}34-259-extVD4^{D}-extVD4^{E}-extVD4^{F}-extVD4^{G}	Emulsified in cationic adjuvant formulation 1 (CAF01)	B6C3F1 and C3H/HeN mice (6-8 weeks)<br>3x s.c. & i.n.; 5 µg antigen; 2-week interval<br>Challenge: i.n. with 1x10^6 IFUs of Ct SvD / i.vag with 4-8x10^5 IFUs of Ct SvD or 1x10^6 IFUs of Ct SvF 6 weeks after final vaccination	Strong immune responses to serovar-specific epitopes, the conserved LNPTIAG epitope and neutralized SvD, E and F. Hirep constructs both amplify and broadens antibody responses.<br><br>Protection of mice: reduced load in vagina and prevention of pathological changes in upper genital tract thus controlling ascending infection.<br><br>Adoptive transfer of serum with high-titered neutralizing antibodies protect recipient mice against primary challenge.	Comparison to rMOMP

	With extVD4^D = MOMP^{D}260-327 and extVD4^G = MOMP^{G}261-329<br><br>(cysteines exchanged with serines)			T-cell depletion experiments demonstrate dominant role for CD4+ T cells (multifunctional Th1/Th17 cells) after CAF01 immunization.	
[23] Boje – Follmann 2016<br><br>PROTEIN VACCINE - NANOPARTICLE	Hirep1 = extVD4^D-extVD4^E-extVD4^F<br>CTH93 = CT043 – CT414$_{605-804}$ – MOMP$^D_{34-371}$<br><br>*Designed to promote neutralizing antibodies or cell-mediated immunity, respectively.*	Emulsified in cationic adjuvant formulation 1 (CAF01)<br><br>(+ 9% sucrose, isotonic solution)	Sexually mature female Göttingen minipigs (5-6 months)<br>2x i.m. 10 µg Hirep1+CTH93/CAF01 / 100 µg Hirep1+CTH93/CAF01 / 125 µg UV-SvD/CAF01; 3-week interval<br>Challenge: i.vag at most cranial part of vagina with 3.9*10^9 IFUs of Ct SvD in SPG at week 7 (d30/d32 after last imm)	Hirep1+CTH93/CAF01 vaccine proved highly immunogenic and equally protective as UV-SvD/CAF01 against vaginal SvD infection.<br><br>Strong CMI response.<br><br>High titers of serum and vaginal IgG antibodies, regardless of the dose, particularly against VD4 region. In vitro neutralization of Ct SvD and SvF, consistent in all animals, unlike for UV-SvD immunized animals.<br><br>Individual antigens were recognized with a broad spectrum of B- and T-cell epitopes.	Comparison with UV-inactivated Ct SvD.
[24] Hadad – Andersson 2016<br><br>PROTEIN VACCINE	MOMP VD2/4 chimeric construct (SvE)<br><br>Highly immunogenic sequences of MOMP, minimizing presence of hydrophobic regions, and spacing with flexible amino acid sequence.	Cholera toxin (CT) / cholera toxin subunit A1 (CTA1-DD)	C57BL/6 (H-2b) and C3H/HeN (H-2k) mice (6-8 weeks)<br>3x i.n. 10 µg construct + 1 µg CT + 1x i.vag 10 µg construct (+ 2 µg CT); 7-10 day interval<br>Challenge: 3*10^4 IFUs of Ct SvD	Significantly less bacterial shedding and better protected against infertility.<br>Both systemic (IgG1 and IgG2c) and local (IgA) antibody responses. Primed T cells producing IFN-γ (Th1), IL-13 (Th2) and IL-17 (Th17) upon intranasal immunization.	
[25] Jiang – Zhang 2017<br><br>PROTEIN VACCINE – CONJUGATE - NANOPARTICLE	SYFPEITHI program<br>MOMP$_{370-387}$<br>MOMP$_{261-276}$<br>MOMP$_{70-81}$<br>1 / 1+2 / 1+2+3 (multi)-epitope	Hepatitis B virus core antigen (HBcAg) – inserted into major immunodominant region (MIR)<br><br>To generate broad immune response<br><br>HBsAg has the capacity to self-assemble into empty virus-like particles (VLPs) in eukaryotic cells – used as HBV vaccine worldwide (Zhu, 2013)	BALB/c mice (H2-Kd)<br>3x s.c. with 100 µg; 2-week interval<br>Challenge: at last day week 8, i.vag, 10^6 IFUs of Ct SvE	Th1 IFN-gamma and Th2 IL-4 sig increased in blood<br>Ct-specific serum IgG and sIgA in vaginal secretions sig elevated<br>Cytotoxicity of splenic lymphocytes sig higher<br><br>Lower IFU number in vaginal swabs and sig shorter time to resolution (18 days to 27)<br>HBcAg/epitope(1+2+3) showed nearly normal oviduct lumens	Results of the HBcAg/epitope(1+2+3) were often best, moreover were often as good or even slightly better than for positive control group (immunized with inactivated EB)<br><br>Tandem Ct MOMP multi-epitopes more effective than single epitope.
[26] Kuczkowska – Dietrich	Hirep2 = extVD4^D-extVD4^E-extVD4^F-extVD4^G	Soluble (H2), emulsified in cationic adjuvant	B6C3F1 mice (6-8 weeks)	H2-displaying L. plantarum evoked cellular response (increased secretion of IFN-γ in	

2017<br><br>PROTEIN VACCINE – (CONJUGATE – LIVE VECTOR) – (NANOPARTICLE)		formulation 1 (CAF01), or anchored to surface of *Lactobacillus plantarum* using N-terminal lipoprotein anchor Lp_1261 (Lp_H2)	1x s.c. 5 µg soluble H2/CAF01; d1 + 2x i.n. 10^9 CFU H2-displaying L. plantarum; d22,d23<br><br>H2 group (only s.c.); H2/Lp_H2 group (all imm); Lp_H2 group (only i.n.)	both splenocytes and PBMC) in mice primed by s.c. H2 immunization.<br><br>Serum IgG response in groups with parenteral vaccination. Serum and mucosal IgA response only in i.n. boosted groups, however low levels.	
[27] Olsen – Andersen 2017<br><br>PROTEIN VACCINE - NANOPARTICLE	Hirep1 = extVD4^D-extVD4^E-extVD4^F<br>ExtVD4^F*4 = MOMP$^F_{261-329}$*4	Emulsified in cationic adjuvant formulation 1 (CAF01)	B6C3F1 mice (6-8 weeks) 3x s.c. / s.c. + i.n.; 5 µg/dose/route; 2-week interval<br>Challenge: i.vag with 1*10^6 IFUs Ct SvF 6 weeks after final immunization / i.vag with 4*10^3 IFUs Ct SvF 72 weeks after final immunization	Similar levels of antibodies and cell-mediated immune responses for both vaccine constructs, but different fine specificities of B cell epitopes. Hirep1 induced a strong response toward neutralizing epitope LNPTIAG, extVD4^F*4 skewed response to a non-neutralizing epitope slightly upstream in the sequence.<br>Vaccination with Hirep1 as opposed to extVD4^F*4 induced significant protection against infection in mice both in short- and long-term vaccination experiments.<br>Passive immunization of Rag1 knockout mice (producing no mature T and B cells) with Hirep1 antibodies completely prevented the establishment of infection in 48% of mice.	
[28] Wang – Zhang 2017<br><br>DNA VACCINE – (CONJUGATE + NANOPARTICLE)	MOMP multi-epitope GDNENQSTVKTNSVPNMSLD QSVVELYTWQASLALSYRLNMF TPYIGV MOMP$_{138-165}$ + MOMP$_{249-268}$	pcDNA3.1-MOMP-HPV16L2/ pcDNA3.1-MOMP/ pcDNA3.1-HPV16L2	BALB/c mice<br>4 x i.m. 100 µg plasmid; week 1, 3, 5 and 7.<br>Challenge: i.vag with 10^6 IFUs Ct SvE; 3 weeks after final immunization	Higher anti-Ct serum IgG (also anti-HPV16L2) and vaginal IgA titers, higher Ct-specific CTL activity and higher level of genital protection (reduced colonization and shorter infection) for chimeric construct.<br>Histochemical staining indicated that genital tract pathologies were eliminated by vaccination with chimeric construct.	
[29] Wern – Follmann 2017<br><br>PROTEIN VACCINE - NANOPARTICLE	CTH522 = MOMP^{D}34-259-extVD4^D-extVD4^E-extVD4^F-extVD4^G<br>Hirep1 = extVD4^D-extVD4^E-extVD4^F<br>CTH93 = CT043 – CT414$_{605-804}$ – MOMP$^D_{34-371}$	Emulsified in cationic adjuvant formulation 1 (CAF01)	B6C3F1 mice (6-8 weeks) s.c. / i.n.; 5 µg CTH522 / 2.5 µg Hirep1 + 2.5 µg CTH93; 2-week interval<br>Challenge: i.vag with 4-8x10^5 IFUs Ct SvD in SPG; 5 weeks after last immunization.<br><br>SC: 2x/3x s.c.<br>IN: 2x i.n.<br>SIM: 2x/3x (s.c. + i.n.)<br>PB: 3x s.c. + 2x i.n.	Similar IFN-γ and IL-17 producing splenocytes (Th1/Th17) and serum IgG1 levels for SC, SIM and PB vaccine strategies. Increased Th1/Th17 responses in the airways, which disseminate to the GT although in low numbers for SIM and PB immunization.<br>SIM and PB protocol also let to IgA responses and increased B cells in lung parenchyma, and in lower numbers in the genital tract.<br>Following vaginal challenge, SIM immunizations gave rise to early IgA response and IgA-secreting plasma cells, but no faster recruitment of T cells.<br>No improved efficacy against infection for SIM in comparison to SC immunization.	Assessment whether simultaneous parenteral (s.c.) and mucosal (i.n.) immunization (SIM) can be done to enable two-visit strategy.

[30] Rose – Foged 2018<br><br>PROTEIN VACCINE - NANOPARTICLE	CTH522 = MOMP^{D}34-259-extVD4^{D}-extVD4^{E}-extVD4^{F}-extVD4^{G}	Glycol chitosan (GC) coated PLGA/DDA/TDB lipid-polymer hybrid nanoparticles (LPNs)	C57BL/6 mice (7-9 weeks)<br>4x i.n. with 5 µg CTH522/CAF01 or 5 µg CTH522/GC-coated LPNs; 2-/3-week interval	Significantly higher serum IgG responses, tendency toward increased serum and vaginal IgA responses (2/5 mice). Equal levels for IFN-γ producing CD4+ T cells in spleen.	Attempt to improve mucosal adjuvant properties liposomes.
[31] Verma – Dennis 2018<br><br>PROTEIN VACCINE - NANOPARTICLE	M278 peptide = MOMP$_{278\text{-}370}$ (fragment containing T-cell epitopes)	Encapsulated in PLA-PEG nanoparticles (PLA-PEG-M278, PPM), self-adjuvanting, biodegradable, co-polymeric	BALB/c mice (4-6 weeks)<br>3x s.c. 50 µg PPM / M278 peptide; 2-week interval<br>Challenge: i.vag with 1*10^5 IFUs of Cm in SPG 3 weeks after last immunization	Significant protection against homologous challenge (reduced bacterial load; as opposed to the M278 peptide immunized mice), correlated with immune responses predominated by IFN-γ, CD4+ T cell proliferation and their differentiation to CD4+ memory and effector T cell phenotypes.<br>Elevated serum antibodies (IgG1 > IgG2b > IgG2a) indicate Th2-directed response, which skewed to a Th1-dominated antibody response after challenge. Elevated mucosal IgG1 and IgA antibody titers followed by increase in IgA after challenge. In vitro neutralization of Cm.	
[32] Nguyen – Dietrich 2021<br><br>PROTEIN VACCINE - NANOPARTICLE	CTH522 = MOMP^{D}34-259-extVD4^{D}-extVD4^{E}-extVD4^{F}-extVD4^{G}	Emulsified in cationic adjuvant formulation 1 (CAF01)	B6C3F1 mice (6-8 weeks)<br>s.c. / i.u. with 5 µg; 3x 2-week interval; only s.c. / only i.u. / SIM (s.c. + i.u.)<br>Challenge: transcervically, directly into uterine horn lumen, with Ct SvD	Vaccine-induced resident memory led to an advantage in response against the first infection, but not the second infection.<br>SIM strategy superior in generation GT tissue-resident Th1/Th17 immunity: faster and stronger IFN-γ/TNF-α Th1, IFN-γ/TNF-α/IL-17 Th17 and innate responses post infection. Significant reduction in bacterial levels.<br>Parenteral strategy generated this GT tissue-resident Th1/Th17 immunity post infection. After second infection, both groups showed similar boost in vaginal IgA and IgG titers.	Suggesting parenteral vaccine strategy is suitable for Ct infections.
[33] Olsen – Follmann 2021<br><br>PROTEIN VACCINE – (NANOPARTICLE)	ExtVD1^{A}*4<br>ExtVD1^{J}*4	For s.c. vaccinations antigens were emulsified in cationic adjuvant formulation 1 (CAF01); i.n. vaccinations unadjuvanted	A/J and C3H/HeN mice (6-8 weeks)<br>3x s.c. or 1st s.c., 2nd s.c. + i.n., 3rd s.c. + i.n. (SIM); 10 µg; 2-week interval<br>Challenge: 6 weeks after final immunization, i.vag, 10^6 Ct SvA (ocular)/HAR-13 or SvIa (genital strain)	Strong (in vitro) cross-neutralizing activity of the ExtVD1^{A}*4 vaccine (good SvA, C, I, Ia, J and K; lower SvB, D, E, F; no SvG and SvH)<br>Sig protection of extVD1^{A}*4/ CAF01 vaccinated mice against challenge with Ct SvA **and SvIa**<br>Strong IgG and IgA responses in genital tract<br>Protective role of extVD1^{A}*4-specific antibodies clear by in vivo neutralization exp<br>T-cell epitopes in conserved N-terminal part of extVD1^{A}*4	Future perspective to combine extVD1^{A}*4 with CTH522 (contains extVD4 repeat, protective against other serovars SvD, E, F, G, K, B, L1, L2, L3)
[34] Collar – Frietze 2022	Conserved neutralizing epitope in VD4 TTLNPTIAG / TTLNPTIAGA	Expressed on coat protein of MS2 bacteriophage VLP in constrained, surface-exposed AB loop	BALB/c mice<br>3x i.m. 5 µg VLP; 3-week interval	Anti-peptide serum IgG at 10^4 endpoint dilution titer. In subset of mice detectable IgG in the genital tract. IgG binds Ct EBs.	

PROTEIN VACCINE – LIVE BACTERIOPHAGE VECTOR			Challenge: i.vag with 2*10^4 IFUs of luciferase-expressing Cm in SPG; d66	Protection to vaginal Cm infection (bacterial burden assessed by in vivo imaging system) better for MS2-TTLNPTIAG vaccine (1.02 log decrease in mean burden).	
[35] Garcia-del Rio – Landin<br>2022<br><br>PROTEIN VACCINE - NANOPARTICLE	CTH522 = MOMP^{D}34-259-extVD4^{D}-extVD4^{E}-extVD4^{F}-extVD4^{G}	Emulsified in cationic adjuvant formulation 1 (CAF01) / ternary optimized hydrogel (OGEL)	CB6F1 mice (7-9 weeks)<br>1x s.c. with 5 µg CTH522/CAF01 / s.l. with 2 µg CTH522 (+ 2x s.l. with 2 µg CTH522 (/OGEL); d0, d15, d35	Parenteral-sublingual prime-boost immunization, using CAF01 and OGEL as CTH522 carriers, shows tendency to increase Th1/Th17 responses (in spleen and cervical lymph nodes) when compared to mucosal or parenteral vaccination alone. Also local IgA response in genital tract (and serum).	Sublingual boosting with vaccine-loaded mucoadhesive thermosensitive hydrogel posterior to systemic priming.
[36] Lorenzen – Dietrich<br>2022<br><br>PROTEIN VACCINE – NANOPARTICLE / VIRAL VECTOR<br><br>DNA VACCINE	CTH522 = MOMP^{D}34-259-extVD4^{D}-extVD4^{E}-extVD4^{F}-extVD4^{G}<br>Consensus MOMP antigen (Con E).	CTH522 adjuvanted with CAF01 or AlOH, unadjuvanted for i.n. administration<br><br>ConE delivered by:<br>- Human Adenovirus serotype 5 vector pAL1112 (HuAd5-MOMP)<br>- Modified vaccinia Ankara MVA pox vector p3186 (MVA-MOMP)<br>- Plasmid pcDNA3.1 (DNA-MOMP)	Cynomolgus macaques (*Macaqua fascicularis*)<br>85 µg CTH522 i.m. / 30 µg CTH522 i.n. / 1 mg DNA-MOMP / 10^11 viral particles HuAd5-MOMP / 4*10^8 PFU MVA-MOMP<br>1) 3x i.m. CTH522/AlOH + 2x i.n. CTH522<br>2) 3x i.m. CTH522/CAF01 + 2x i.n. CTH522<br>3) 3x i.m. CTH522/CAF01<br>4) 3x i.d./e.p. DNA-MOMP + 1x i.m. CTH522/CAF01<br>5) 1x i.d./e.p. DNA-MOMP + 1x i.m. HuAd5-MOMP + 1x i.m. MVA-MOMP + 1x i.m. CTH522/CAF01<br>Challenge: at vagino-cervical transition with 5*10^7 IFUs Ct SvD	All constructs raised significant systemic IgG responses, in particular CTH522 protein vaccination. B cell epitopes near or within VD1, VD3 and VD4, with consistent VD4 responses. In vitro neutralization of SvD, SvE and SvF. Vaginal and ocular mucosa showed detectable levels of IgG. Intranasal boosting with unadjuvanted CTH522 had no boosting effect on mucosal antibody levels.<br>In general, CTH522 vaccinated groups established a multifunctional CD4 T cell response, whereas DNA and vector vaccinated groups also established a CD8 T cell response.<br>Several groups showed accelerated clearance of infection, especially DNA group boosted with CAF01 adjuvanted CTH522.	Evaluation of immunization strategies in a NHP model.
[37] Carlsen – Boisen<br>2023<br><br>PROTEIN VACCINE - NANOPARTICLE	CTH522 = MOMP^{D}34-259-extVD4^{D}-extVD4^{E}-extVD4^{F}-extVD4^{G}	Emulsified in cationic adjuvant formulation 1 (CAF01) (s.c.)<br><br>α-GalCer, c-di-GMP, cholera toxin B adjuvant + Microcontainers (MCs) with EL100-55, PLGA or chitosan coating (oral)	CB6F1 mice<br>1x s.c. 5 µg CTH522/CAF01 (+ 2x orally 10 µg CTH522; 3-week interval)	CTH522 formulated with α-GalCer, loaded into MCs with EL100-55, showed to be the most efficient combination for the oral vaccine.<br>Mice receiving oral prime and boosters did show significantly enhanced mucosal immune responses.<br>Mice orally boosted following s.c. prime demonstrated improved systemic and local Th17 responses, and increased local IFN-γ	Procedure to lyophilize vaccine without degrading antigen or losing immunogenicity developed.

				and IgA levels (compared to s.c. prime alone).<br>Mice is not a suitable animal model, MCs are proportionally too large for their GI tract (extremely narrow absorption window).	
[38] Olsen – Follmann 2024<br><br>PROTEIN VACCINE – NANOPARTICLE	CTH522 = $MOMP^{D}$34-259-extVD4^{D}-extVD4^{E}-extVD4^{F}-extVD4^{G}	Emulsified in cationic adjuvant formulation 1 (CAF01)	B6C3F1/OlaHsd (H-2b,k) and C3H/HeNHsd (H-2k) mice (6-8 weeks)<br>3x s.c. 10-25 µg (+ 1x i.n. 25 µg unadjuvanted); 2-week interval<br>Challenge: i.vag with 1.5*10^3-1*10^5 IFUs Ct SvD in SPG	CTH522 specific immune responses generated in mouse model are, to a high degree, translatable to humans regarding specificity and functionality of the antibody response and the cytokine profile of the CMI response.<br>The common immune signature protected mice against ascending infection and pathology, and is sustained and protective for up to 1-year.	Comparative immunological characterization of CTH522/CAF01 in female mice and humans.<br>Proposal for phase IIb study.

1. Toye, B.; Zhong, G.; Peeling, R.; Brunham, R.C. Immunologic Characterization of a Cloned Fragment Containing the Species-Specific Epitope from the Major Outer Membrane Protein of Chlamydia Trachomatis. *Infect Immun* **1990**, *58*, 3909–3913, doi:10.1128/iai.58.12.3909-3913.1990.

2. Hayes, L.J.; Conlan, J.W.; Everson, J.S.; Ward, M.E.; Clarke, I.N. Chlamydia Trachomatis Major Outer Membrane Protein Epitopes Expressed as Fusions with LamB in an Attenuated AroA Strain of Salmonella Typhimurium; Their Application as Potential Immunogens. *J Gen Microbiol* **1991**, *137*, 1557–1564, doi:10.1099/00221287-137-7-1557.

3. Cheng, X.; Pal, S.; de la Maza, L.M.; Peterson, E.M. Characterization of the Humoral Response Induced by a Peptide Corresponding to Variable Domain IV of the Major Outer Membrane Protein of Chlamydia Trachomatis Serovar E. *Infect Immun* **1992**, *60*, 3428–3432.

4. Tuffrey, M.; Alexander, F.; Conlan, W.; Woods, C.; Ward, M. Heterotypic Protection of Mice against Chlamydial Salpingitis and Colonization of the Lower Genital Tract with a Human Serovar F Isolate of Chlamydia Trachomatis by Prior Immunization with Recombinant Serovar L1 Major Outer-Membrane Protein. *J Gen Microbiol* **1992**, *138*, 1707–1715, doi:10.1099/00221287-138-8-1707.

5. Murdin, A.D.; Su, H.; Manning, D.S.; Klein, M.H.; Parnell, M.J.; Caldwell, H.D. A Poliovirus Hybrid Expressing a Neutralization Epitope from the Major Outer Membrane Protein of Chlamydia Trachomatis Is Highly Immunogenic. *Infect Immun* **1993**, *61*, 4406–4414, doi:10.1128/iai.61.10.4406-4414.1993.

6. Su, H.; Caldwell, H.D. Immunogenicity of a Synthetic Oligopeptide Corresponding to Antigenically Common T-Helper and B-Cell Neutralizing Epitopes of the Major Outer Membrane Protein of Chlamydia Trachomatis. *Vaccine* **1993**, *11*, 1159–1166, doi:10.1016/0264-410x(93)90080-h.

7. Qu, Z.; Cheng, X.; de la Maza, L.M.; Peterson, E.M. Analysis of the Humoral Response Elicited in Mice by a Chimeric Peptide Representing Variable Segments I and IV of the Major Outer Membrane Protein of Chlamydia Trachomatis. *Vaccine* **1994**, *12*, 557–564, doi:10.1016/0264-410x(94)90317-4.

8. Knight, S.C.; Iqball, S.; Woods, C.; Stagg, A.; Ward, M.E.; Tuffreyt Antigen, M. A Peptide of Chlamydia Trachomatis Shown to Be a Primary T-Cell Epitope in Vitro Induces Cell-Mediated Immunity in Vivo. *Immunology* **1995**, *85*, 8–15.

9. Su, H.; Pamell, M.; Caldwell, H.D. Protective Efficacy of a Parenterally Administered MOMP-Derived Synthetic Oligopeptide Vaccine in a Murine Model of Chlamydia Trachomatis Genital Tract Infection: Serum Neutralizing IgG Antibodies Do Not Protect against Chlamydial Genital Tract Infection. *Vaccine* **1995**, *13*, 1023–1032, doi:10.1016/0264-410X(95)00017-U.

10. Peterson, E.M.; Cheng, X.; Qu, Z.; de la Maza, L.M. The Effect of Orientation within a Chimeric Peptide of the Immunogenicity of Chlamydia Trachomatis Epitopes. *Mol Immunol* **1996**, *33*, 335–339, doi:10.1016/0161-5890(95)00157-3.

11. Motin, V.L.; de la Maza, L.M.; Peterson, E.M. Immunization with a Peptide Corresponding to Chlamydial Heat Shock Protein 60 Increases the Humoral Immune Response in C3H Mice to a Peptide Representing Variable Domain 4 of the Major Outer Membrane Protein of Chlamydia Trachomatis. *Clin Diagn Lab Immunol* **1999**, *6*, 356–363, doi:10.1128/cdli.6.3.356-363.1999.

12. He, Q.; Martinez-Sobrido, L.; Eko, F.O.; Palese, P.; Garcia-Sastre, A.; Lyn, D.; Okenu, D.; Bandea, C.; Ananaba, G.A.; Black, C.M.; et al. Live-Attenuated Influenza Viruses as Delivery Vectors for Chlamydia Vaccines. *Immunology* **2007**, *122*, 28–37, doi:10.1111/j.1365-2567.2007.02608.x.

13. Yang, S.R.; Wu, X.L.; Yang, Y.; Sun, J.H.; Lu, J.R.; Yu, Y.L.; Wang, L.Y. Protective Effects of Fusion Protein of Hsp65-MOMP-T-Epitopes on C. Trachomatis Genital Tract Infection of Mice. *Journal of Jilin University Medicine Edition* **2007**, *33*, 440–444.

14. Shi, Z.; Zhu, S.; Xu, W.; Lu, L.; Li, L.; Zhang, L. The Cellular Immune Response Produced in BALB/c Mice Immunized with HPV6b L1/Ct MOMP Multi-Epitope Chimeric DNA . *Chinese Journal of Microbiology and Immunology* **2010**, *30*, 942–948.

15. Taha, M.A.; Singh, S.R.; Hulett, K.; Pillai, S.R.; Agee, R.; Dennis, V.A. A Peptide Containing T-Cell Epitopes of Chlamydia Trachomatis Recombinant MOMP Induces Systemic and Mucosal Antibody Responses in Mice. *World J Vaccines* **2011**, *01*, 138–147, doi:10.4236/wjv.2011.14014.

16. Xu, W.; Liu, J.; Gong, W.; Chen, J.; Zhu, S.; Zhang, L. Protective Immunity against Chlamydia Trachomatis Genital Infection Induced by a Vaccine Based on the Major Outer Membrane Multi-Epitope Human Papillomavirus Major Capsid Protein L1. *Vaccine* **2011**, *29*, 2672–2678, doi:10.1016/j.vaccine.2010.12.132.

17. Zhu, S.; Feng, Y.; Rao, P.; Xue, X.; Chen, S.; Li, W.S.; Zhu, G.; Zhang, L. Hepatitis B Virus Surface Antigen as Delivery Vector Can Enhance Chlamydia Trachomatis MOMP Multi-Epitope Immune Response in Mice. *Appl Microbiol Biotechnol* **2014**, *98*, 4107–4117, doi:10.1007/s00253-014-5517-x.

18. Dixit, S.; Singh, S.R.; Yilma, A.N.; Agee, R.D.; Taha, M.; Dennis, V.A. Poly(Lactic Acid)-Poly(Ethylene Glycol) Nanoparticles Provide Sustained Delivery of a Chlamydia Trachomatis Recombinant MOMP Peptide and Potentiate Systemic Adaptive Immune Responses in Mice. *Nanomedicine* **2014**, *10*, 1311–1321, doi:10.1016/j.nano.2014.02.009.

19. Tu, J.; Hou, B.; Wang, B.; Lin, X.; Gong, W.; Dong, H.; Zhu, S.; Chen, S.; Xue, X.; Zhao, K.N.; et al. A Multi-Epitope Vaccine Based on Chlamydia Trachomatis Major Outer Membrane Protein Induces Specific Immunity in Mice. *Acta Biochim Biophys Sin (Shanghai)* **2014**, *46*, 401–408, doi:10.1093/abbs/gmu016.

20. Jiang, P.; Du, W.; Xiong, Y.; Lv, Y.; Feng, J.; Zhu, S.; Xue, X.; Chen, S.; Zhang, L. Hepatitis B Virus Core Antigen as a Carrier for Chlamydia Trachomatis MOMP Multi-Epitope Peptide Enhances Protection against Genital Chlamydial Infection. *Oncotarget* **2015**, *6*, 43281–43292, doi:10.18632/oncotarget.6533.

21. Lorenzen, E.; Follmann, F.; Bøje, S.; Erneholm, K.; Olsen, A.W.; Agerholm, J.S.; Jungersen, G.; Andersen, P. Intramuscular Priming and Intranasal Boosting Induce Strong Genital Immunity through Secretory IgA in Minipigs Infected with Chlamydia Trachomatis. *Front Immunol* **2015**, *6*, doi:10.3389/fimmu.2015.00628.

22. Olsen, A.W.; Follmann, F.; Erneholm, K.; Rosenkrands, I.; Andersen, P. Protection Against Chlamydia Trachomatis Infection and Upper Genital Tract Pathological Changes by Vaccine-Promoted Neutralizing Antibodies Directed to the VD4 of the Major Outer Membrane Protein. *Journal of Infectious Diseases* **2015**, *212*, 978–989, doi:10.1093/infdis/jiv137.

23. Bøje, S.; Olsen, A.W.; Erneholm, K.; Agerholm, J.S.; Jungersen, G.; Andersen, P.; Follmann, F. A Multi-Subunit Chlamydia Vaccine Inducing Neutralizing Antibodies and Strong IFN-γ + CMI Responses Protects against a Genital Infection in Minipigs. *Immunol Cell Biol* **2016**, *94*, 185–195, doi:10.1038/icb.2015.79.

24. Hadad, R.; Marks, E.; Kalbina, I.; Schön, K.; Unemo, M.; Lycke, N.; Strid, Å.; Andersson, S. Protection against Genital Tract Chlamydia Trachomatis Infection Following Intranasal Immunization with a Novel Recombinant MOMP VS2/4 Antigen. *APMIS* **2016**, *124*, 1078–1086, doi:10.1111/apm.12605.

25. Jiang, P.; Cai, Y.; Chen, J.; Ye, X.; Mao, S.; Zhu, S.; Xue, X.; Chen, S.; Zhang, L. Evaluation of Tandem Chlamydia Trachomatis MOMP Multi-Epitopes Vaccine in BALB/c Mice Model. *Vaccine* **2017**, *35*, 3096–3103, doi:10.1016/j.vaccine.2017.04.031.

26. Kuczkowska, K.; Myrbråten, I.; Øverland, L.; Eijsink, V.G.H.; Follmann, F.; Mathiesen, G.; Dietrich, J. Lactobacillus Plantarum Producing a Chlamydia Trachomatis Antigen Induces a Specific IgA Response after Mucosal Booster Immunization. *PLoS One* **2017**, *12*, doi:10.1371/journal.pone.0176401.

27. Olsen, A.W.; Lorenzen, E.K.; Rosenkrands, I.; Follmann, F.; Andersen, P. Protective Effect of Vaccine Promoted Neutralizing Antibodies against the Intracellular Pathogen Chlamydia Trachomatis. *Front Immunol* **2017**, *8*, doi:10.3389/fimmu.2017.01652.

28. Wang, L.; Cai, Y.; Xiong, Y.; Du, W.; Cen, D.; Zhang, C.; Song, Y.; Zhu, S.; Xue, X.; Zhang, L. DNA Plasmid Vaccine Carrying Chlamydia Trachomatis (Ct) Major Outer Membrane and Human Papillomavirus 16L2 Proteins for Anti-Ct Infection. *Oncotarget* **2017**, *8*, 33241–33251, doi:10.18632/oncotarget.16601.

29. Wern, J.E.; Sorensen, M.R.; Olsen, A.W.; Andersen, P.; Follmann, F. Simultaneous Subcutaneous and Intranasal Administration of a CAF01-Adjuvanted Chlamydia Vaccine Elicits Elevated IgA and Protective Th1/Th17 Responses in the Genital Tract. *Front Immunol* **2017**, *8*, doi:10.3389/fimmu.2017.00569.

30. Rose, F.; Wern, J.E.; Gavins, F.; Andersen, P.; Follmann, F.; Foged, C. A Strong Adjuvant Based on Glycol-Chitosan-Coated Lipid-Polymer Hybrid Nanoparticles Potentiates Mucosal Immune Responses against the Recombinant Chlamydia Trachomatis Fusion Antigen CTH522. *Journal of Controlled Release* **2018**, *271*, 88–97, doi:10.1016/j.jconrel.2017.12.003.

31. Verma, R.; Sahu, R.; Dixit, S.; Duncan, S.A.; Giambartolomei, G.H.; Singh, S.R.; Dennis, V.A. The Chlamydia M278 Major Outer Membrane Peptide Encapsulated in the Poly(Lactic Acid)-Poly(Ethylene Glycol) Nanoparticulate Self-Adjuvanting Delivery System Protects Mice against a Chlamydia Muridarumgenital Tract Challenge by Stimulating Robust Systemic and Local Mucosal Immune Responses. *Front Immunol* **2018**, *9*, doi:10.3389/fimmu.2018.02369.

32. Nguyen, N.D.N.T.; Guleed, S.; Olsen, A.W.; Follmann, F.; Christensen, J.P.; Dietrich, J. Th1/Th17 T Cell Tissue-Resident Immunity Increases Protection, But Is Not Required in a Vaccine Strategy Against Genital Infection With Chlamydia Trachomatis. *Front Immunol* **2021**, *12*, doi:10.3389/fimmu.2021.790463.

33. Olsen, A.W.; Rosenkrands, I.; Holland, M.J.; Andersen, P.; Follmann, F. A Chlamydia Trachomatis VD1-MOMP Vaccine Elicits Cross-Neutralizing and Protective Antibodies against C/C-Related Complex Serovars. *NPJ Vaccines* **2021**, *6*, doi:10.1038/s41541-021-00312-9.

34. Collar, A.L.; Linville, A.C.; Core, S.B.; Frietze, K.M. Epitope-Based Vaccines against the Chlamydia Trachomatis Major Outer Membrane Protein Variable Domain 4 Elicit Protection in Mice. *Vaccines (Basel)* **2022**, *10*, doi:10.3390/vaccines10060875.

35. Garcia-del Rio, L.; Diaz-Rodriguez, P.; Pedersen, G.K.; Christensen, D.; Landin, M. Sublingual Boosting with a Novel Mucoadhesive Thermogelling Hydrogel Following Parenteral CAF01 Priming as a Strategy Against Chlamydia Trachomatis. *Adv Healthc Mater* **2022**, *11*, doi:10.1002/adhm.202102508.

36. Lorenzen, E.; Contreras, V.; Olsen, A.W.; Andersen, P.; Desjardins, D.; Rosenkrands, I.; Juel, H.B.; Delache, B.; Langlois, S.; Delaugerre, C.; et al. Multi-Component Prime-Boost Chlamydia Trachomatis Vaccination Regimes Induce Antibody and T Cell Responses and Accelerate Clearance of Infection in a Non-Human Primate Model. *Front Immunol* **2022**, *13*, doi:10.3389/fimmu.2022.1057375.

37. Carlsen, P.H.R.; Kjeldsen, R.B.; Pedersen, G.K.; Christensen, D.; Nielsen, L.H.; Boisen, A. Oral Vaccination Using Microdevices to Deliver α-GalCer Adjuvanted Vaccine Afford a Mucosal Immune Response. *Journal of Controlled Release* **2023**, *353*, 134–146, doi:10.1016/j.jconrel.2022.11.015.

38. Olsen, A.W.; Rosenkrands, I.; Jacobsen, C.S.; Cheeseman, H.M.; Kristiansen, M.P.; Dietrich, J.; Shattock, R.J.; Follmann, F. Immune Signature of Chlamydia Vaccine CTH522/CAF®01 Translates from Mouse-to-Human and Induces Durable Protection in Mice. *Nat Commun* **2024**, *15*, doi:10.1038/s41467-024-45526-2.
